# Climate Change Impacts on Greenhouse Horticulture in the Mediterranean Basin: Challenges and Adaptation Strategies

**DOI:** 10.3390/plants14213390

**Published:** 2025-11-05

**Authors:** Dimitrios Fanourakis, Georgios Tsaniklidis, Theodora Makraki, Nikolaos Nikoloudakis, Thomas Bartzanas, Leo Sabatino, Hicham Fatnassi, Georgia Ntatsi

**Affiliations:** 1 Laboratory of Quality and Safety of Agricultural Products, Landscape and Environment, Department of Agriculture, School of Agricultural Sciences, Hellenic Mediterranean University, Estavromenos, 71004 Heraklion, Greece; makraki.th@gmail.com; 2 Institute of Olive Tree, Subtropical Plants and Viticulture, Hellenic Agricultural Organization ‘ELGO-Dimitra’, Kastorias 32A, 71307 Heraklion, Greece; tsaniklidis@elgo.gr; 3 Department of Agricultural Science, Biotechnology and Food Science, Cyprus University of Technology, Limassol 3036, Cyprus; n.nikoloudakis@cut.ac.cy; 4 Farm Structures Lab, Department of Natural Resources and Agricultural Engineering, Agricultural University of Athens, 11855 Athens, Greece; t.bartzanas@aua.gr; 5 Dipartimento di Scienze Agrarie, Alimentari e Forestali, University of Palermo, Viale delle Scienze, Ed. 4, 90128 Palermo, Italy; leo.sabatino@unipa.it; 6 INRAE, UR 1115 Plantes et Systèmes de Culture Horticoles, 84000 Avignon, France; hicham.fatnassi@inrae.fr; 7 Laboratory of Vegetable Production, Department of Crop Science, Agricultural University of Athens, 11855 Athens, Greece

**Keywords:** climate change adaptation, crop resilience, cucumber, greenhouse horticulture, Mediterranean zone, sweet pepper, tomato

## Abstract

Greenhouse horticulture is a cornerstone of year-round vegetable production. However, escalating climate change is intensifying abiotic stressors (i.e., elevated temperatures, increased vapor pressure deficits, water shortage, and modified solar radiation), threatening both crop productivity and postharvest performance. This review synthesizes current knowledge on how these climatic shifts impact greenhouse microclimate, pest and disease patterns, energy and water requirements, as well as crop development in the Mediterranean region. This study focuses on three major crops (tomato, cucumber, and sweet pepper), which prevail in the regional protected cultivation sector. Among the climate-induced stressors examined, elevated temperature emerges as the primary environmental constraint on greenhouse productivity. In reality, however, a combination of climate-induced stressors is at play, acting simultaneously and often synergistically. Among crops, cucumber generally displays the highest sensitivity to climate-induced shifts, whereas sweet pepper tends to be the most resilient. Next, adaptive strategies are explored, including precision irrigation, structural retrofitting measures, renewable energy integration, Decision Support Systems, and climate-resilient cultivars. Regional case studies revealed diverse country-specific counteractive innovations. As key elements of inclusive climate adaptation, supportive policy frameworks and a practical agenda of targeted research priorities are outlined. In conclusion, the sustainability of greenhouse horticulture under a changing climate demands integrated, technology-driven, and region-focused approaches.

## 1. Introduction

The Mediterranean basin, including Southern Europe, North Africa, and parts of the Middle East, is commonly acknowledged as a climate change hotspot [1,2]. This zone has a typical semi-arid to sub-humid climate characterized by hot and dry summers along with mild and wet winters. In the Mediterranean zone, the analyses of weather observational records over the past decades indicate longer periods of elevated temperatures, reduced precipitation, extended dry spells, and increased frequency of extreme climatic events [3,4,5,6]. These climatic hazards are projected to occur more often and with greater intensity, carrying substantial implications for natural ecosystems, agricultural systems and protected cultivation [7].

In the Mediterranean, greenhouse horticulture is a vital component of regional agroeconomies, dominated by tomato (*Solanum lycopersicum* L.), cucumber (*Cucumis sativus* L.), and sweet pepper (*Capsicum annuum* L.). Spain, Italy, Greece, and Turkey are the leading producers and exporters of these vegetables, which are cultivated extensively under greenhouse conditions to meet the need for year-round supply and better quality [8]. By providing a semi-controlled environment, protected cultivation buffers the unpredictability of open field conditions to a certain extent. Inevitably, however, the performance of Mediterranean greenhouses is still associated with external climatic conditions, especially in facilities which rely primarily on passive ventilation and have limited cooling capacity [9,10].

As climatic change progresses, heat waves, reduced relative air humidity (RH), elevated vapor pressure deficits (VPDs), altered solar radiation and erratic precipitation patterns increasingly compromise the control of microclimatic factors within the greenhouses [11]. As rainfall declines, greenhouse horticulture is becoming increasingly reliant on groundwater resources, potentially leading to aquifer depletion and associated environmental degradation. These climatic shifts adversely affect yield potential and fruit quality, increase pest and disease risks, as well as amplify water and energy demands [12,13]. In this regard, the greenhouse horticultural industry is increasingly experiencing the need to adapt to unprecedented climate change-induced challenges by developing an array of strategies. Examples include the climate-responsive architecture, resource-efficient cultivation protocols, and cultivar selection based on climate resilience [14,15,16,17].

**Figure 1:**
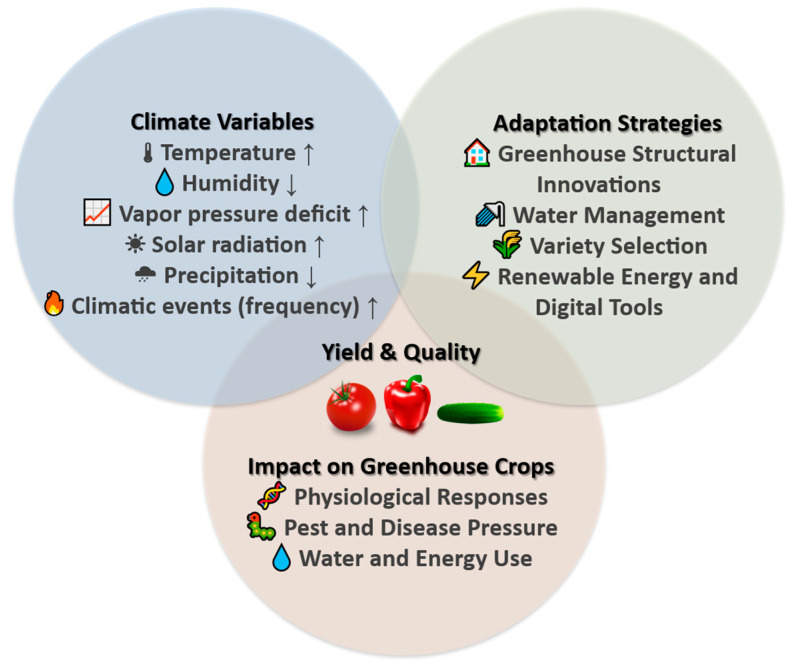
Overview of climate change challenges, impacts, and adaptive strategies in Mediterranean greenhouse horticulture. Extreme climatic events include recurrent heatwaves, prolonged droughts, and intense rainfall episodes, which collectively intensify environmental variability and stress greenhouse production systems. Arrows indicate direction of projected change (↑ increase, ↓ decrease).

In the Mediterranean context, this review addresses the complex interactions between climate change and greenhouse production of three major vegetable crops (tomato, cucumber, and sweet pepper) (Figure 1). It synthesizes current understanding of physiological, agronomic, and postharvest responses to climate change-induced abiotic stresses. This survey further identifies and assesses the comparative effectiveness of innovative approaches for more sustainable and more resilient greenhouse vegetable production under the evolving climatic regime. Emphasis is placed on crop-specific benefits and regional vulnerabilities.

Before presenting climate projections and their implications, it is important to outline the strategy used for retrieving and selecting the literature reviewed in this study. We searched Web of Science, Scopus and ScienceDirect, using combinations of “greenhouse horticulture”, “climate change”, “climate-smart agriculture”, “abiotic stress”, “biotic stress”, “resource-use efficiency”, “renewable energy”, “Decision Support Systems (DSS)”, “tomato”, “cucumber” and “sweet pepper”. Each query was paired with the name of every Mediterranean country. Following FAO/UNEP, the Mediterranean basin includes Albania, Algeria, Bosnia and Herzegovina, Croatia, Cyprus, Egypt, France, Greece, Israel, Italy, Lebanon, Libya, Malta, Monaco, Montenegro, Morocco, Palestine, Slovenia, Spain, Syria, Tunisia and Turkey. Portugal and Jordan were also considered because their agro-climatic conditions closely resemble those of the Mediterranean. All countries were represented in the retrieved literature, with the exception of Monaco, where no relevant studies were found. The primary time window was 2015–2025. When no records were found, we extended the window stepwise to earlier decades. In total, 446 references were retained, most published after 2015, reflecting intensified research on climate-change impacts and adaptation in Mediterranean greenhouse horticulture.

## 2. Climate Projections for the Mediterranean Region (2041–2070 Horizon)

**Figure 2:**
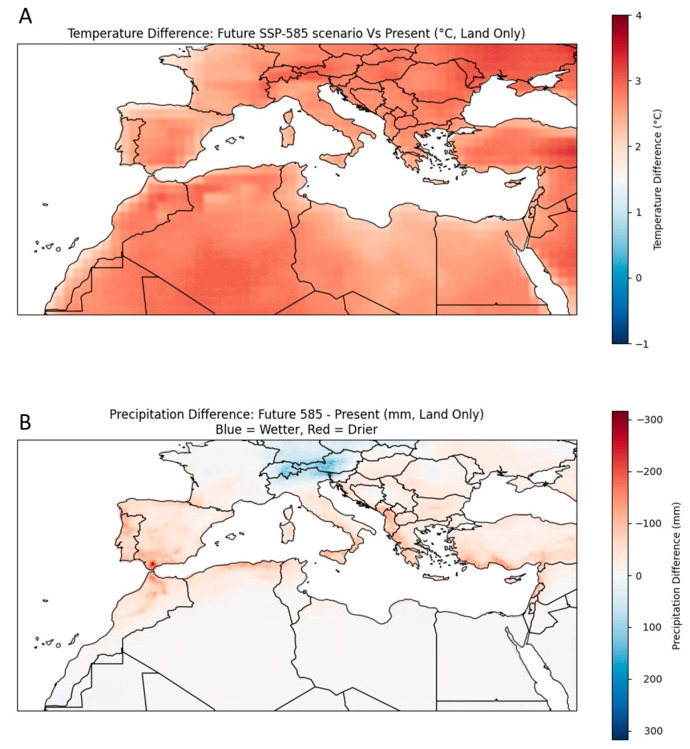
Projected changes in mean annual temperature (**A**) and annual precipitation (**B**) across the Mediterranean basin under the SSP5-8.5 scenario for the mid-century period (2041–2070), based on the GFDL-ESM4 model. Climate variables were derived from the CHELSA V2.1 dataset, using BIO1 (mean annual temperature) and BIO12 (annual precipitation). Baseline data refer to the period 1981–2010. Future changes over terrestrial surfaces were calculated by subtracting present from projected values, using georeferenced raster maps (GeoTIFF format) pre-masked to the study area. Data processing and visualization were performed in Python (v3.13), utilizing the rasterio, numpy, and cartopy libraries for pixel-wise calculations and spatial rendering.

By mid-century, the Mediterranean basin is projected to undergo extensive climatic shifts (Table 1). Based on projections of both regional climate models and the IPCC (Intergovernmental Panel on Climate Change) Sixth Assessment Report (AR6), the Mediterranean zone is expected to warm significantly faster than the global average [6,17]. By considering the intermediate and high-emission scenarios [Shared Socioeconomic Pathways (SSP) 2–4.5 and 5–8.5, respectively], mean annual temperatures are projected to rise by 1.5–2.5 °C by 2041–2070, depending on subregion and altitude [2,6]. Projections further reveal that countries such as Spain and Turkey will experience a larger temperature increase as compared to others, such as Tunisia (Figure 2). Depending on the region, this warming tendency can impose additional physiological stress on greenhouse crops, and challenge existing climate management strategies.

In addition to the temperature mean rise, maximum temperature extremes are projected to develop more frequent and more intense. The number of days exceeding 35 °C is expected to increase prominently, especially during the summer season, contributing to severe heat stress events in protected cultivation systems [18]. Nighttime minimum temperatures are also anticipated to increase by 1.5–2.5 °C, which can disturb plant respiration, sugar metabolism, and hence overall energy balance [2,6,19,20].

Throughout this review, the temperature-related factors (heat stress, high temperature, and thermal extremes) are considered together, without implying that they are interchangeable or equally stressful to plants. Accordingly, the terminology as reported in the cited study is retained, to preserve the original contextual framing. That said, elevated temperature typically corresponds to an increase in VPD, a microclimatic parameter jointly influenced by both air temperature and RH. VPD reflects the air evaporative demand and serves as a principal driver of plant transpiration along with radiation and air velocity [21,22]. In greenhouse environments, elevated VPD is also widely acknowledged as a critical climate change-induced stressor, as it accelerates transpirational water loss and imposes physiological constraints particularly under heat stress [23].

Although RH projections vary by subregion and season, most climate models denote a declining tendency in RH, especially during the summer season, across the Mediterranean zone [18,24]. This decline, in combination with increasing temperatures, results in considerably elevated VPD. High VPD raises the evaporative demand, posing significant constraints on plant water relations and greenhouse climate control systems.

Evapotranspiration (ET) is also projected to increase by 10–15% during warm seasons, owing to higher VPD and enhanced solar radiation. This rise in ET will escalate irrigation demands, adding further pressure on already scarce water resources, especially during the summer period [25,26,27].

Shifts in radiation dynamics are also expected. Solar radiation intensity is projected to increase by 2–8%, mainly due to reduced cloud cover and lower aerosol concentrations [28,29,30]. On a positive note, this could boost photosynthesis under some conditions. However, it may also intensify greenhouse heat load, driving the need for spectral management through shading nets and adaptive lighting systems.

Although a recent study reported that Mediterranean precipitation has remained largely stationary over the long term (1871–2020), it also recorded marked interannual and multi-decadal variability [31]. Therefore, even in the absence of a long-term decline, the prominent interannual and decadal variability can rigorously limit water availability at the timescales most critical for agricultural context. Looking ahead, precipitation patterns are projected to shift towards reduced annual rainfall events (−10 to −20%), mainly in the summer period. Southern Europe and parts of the eastern Mediterranean are expected to be the most severely impacted (ref. [2], see also Figure 2). In combination with increased ET, the reduced precipitation will magnify water scarcity and hydrological drought, further compromising irrigation sustainability.

Notably, the above-mentioned projected climatic changes are not isolated but occur simultaneously combining their individual impacts on greenhouse horticulture [32]. The concurrent rise in temperature, VPD, and radiation, coupled with periodic heatwaves, and declining precipitation amounts presents a multi-stress context. This context often surpasses both the physiological limits of the crop and the capacity of greenhouse systems to buffer climatic extremes. This junction of stressors requires integrated adaptation strategies, addressing the complexity of overlapping climatic hazards, rather than dealing with each variable in isolation.

**Table 1:** Projected climate change indicators in the Mediterranean region by mid-century. RH, relative air humidity; ET, Evapotranspiration; T, temperature; VPD, vapor pressure deficit. Arrows indicate direction of projected change (↑ increase, ↓ decrease).

Climate Variable	Projected Change	Time Horizon	Key References
Mean annual T	↑ 1.5–2.5 °C	2041–2070 (SSP2-4.5/8.5)	[2,6]
Maximum T Extremes	↑ frequency of >35 °C days	2041–2070 (SSP2-4.5/8.5)	[6,18]
Nighttime T	↑ 1.5–2.5 °C	2041–2070 (SSP2-4.5/8.5)	[2]
Heatwave frequency	↑ 2–3×	2041–2070	[33]
RH	↓ 5–10% in summer	2041–2070	[18,24]
VPD	↑ due to ↑ T and ↓ RH	2041–2070	[18,26]
Solar radiation intensity	↑ 2–8% due to reduced cloud cover	2041–2070	[28,34]
ET	↑ 10–15% in warm seasons	2041–2070	[25,26]
Precipitation	↓ 10–20% (especially summer)	2041–2070	[2]

## 3. Impacts on Greenhouse Vegetable Crops

### 3.1. Physiological Responses

In protected cultivation, physiological processes are synergistically regulated by the following microclimatic variables: temperature, RH, light (intensity and spectral composition), and CO_2_ concentration. In the Mediterranean zone (i.e., under increasing temperatures, declining RH, rising VPD, and modified solar radiation), these environmental conditions are often sustained far beyond optimal thresholds, challenging core physiological functions, such as photosynthesis, transpiration, and reproductive development [6,35]. Representative case studies focusing on climate change-induced physiological responses in Mediterranean greenhouse systems are compiled (Supplementary Table S1) and ranked by significance (Table 2).

Among the various stressors, heat, especially during the reproductive phase, has been identified as one of the most detrimental. In tomato and sweet pepper, air temperatures exceeding 32 °C during anthesis adversely affect male gametophyte development, decreasing pollen viability, anther dehiscence, and stigma receptivity, consequently deteriorating fruit set and yield [36,37,38]. Cucumber, characterized by monoecious or gynoecious sex expression, displays high sensitivity to elevated temperature, which alters floral ratios and impairs fruit yield [39].

Photosynthesis is another primary impact of thermal stress. Beyond the 30–32 °C threshold, Rubisco enzyme kinetics become suboptimal, and photorespiration increases, resulting in declining carbon assimilation [40,41]. At the same time, heat-accelerated respiration escalates metabolic demands, lessening net carbon gain [42]. These effects are aggravated under water stress, where stomatal closure owing to elevated VPD further limits internal CO_2_ availability [43]. Regional studies (Spain, Turkey, and Greece) denote consistent drops in photosynthetic rates and associated chlorophyll fluorescence indices (e.g., F_v_/F_m_, PI_ABS_) in tomato and cucumber under thermal extremes [37,44,45]. Under high VPD and water stress, stomatal dynamics also adjust, though not always favorably [46]. Reduced stomatal conductance benefits water conservation, but often at the expense of carbon gain. Regional studies (Italy and Spain) denote lower hydration level and turgor loss in cucumber and pepper under sustained high VPD and soil drying [47,48], emphasizing the compromise between water-use efficiency (WUE) and photosynthetic productivity.

By modifying stratospheric ozone, aerosol loading and cloudiness, climate change can alter the radiation intensity and spectral quality reaching plants, and thus potentially stimulating stress effects. Radiation stress, mainly by short-wavelength radiation [ultraviolet (UV)-B], elicits photoinhibition and pigment degradation [49]. Summer radiation peaks frequently trigger chlorophyll damage, thylakoid disintegration, and weakened electron transport [50,51]. Additionally, accumulation of photoprotective pigments (e.g., anthocyanins, flavonoids) may act antagonistically to chlorophyll biosynthesis, lowering light-harvesting efficiency [52].

Heat stress disturbs source-sink relations too [53]. In tomato, elevated night temperatures promote respiration rates, exhausting carbohydrate pools vital for fruit development, leading to smaller fruits with inferior carbohydrate content (°Brix) and lower firmness [54,55]. In cucumber, high temperature and VPD magnify fruit transpirational water loss, lowering turgor and eventually shelf life [56]. Hormonal and mineral imbalances elicited by inconsistent water flow also promote the development of physiological disorders, such as blossom-end rot (BER), cracking, and sunscald [57,58]. Under high VPD conditions, BER prevalence has been associated with calcium transport disruption during rapid fruit expansion phases.

Oxidative stress seems to be a universal response to combined heat and radiation stress [59,60]. Elevated reactive oxygen species (ROS) production elicits damage to lipids, proteins, and nucleic acids [61,62]. While the response of antioxidant enzymes (e.g., superoxide dismutase, catalase, and ascorbate peroxidase) induce protection [59,63], their activity may be insufficient during prolonged or compound stress [64]. Genotypic differences in antioxidant capacity, as described in tomato and cucumber, provide breeding potential for enhanced resilience [65,66]

Root system plasticity also participates in stress mitigation. Under deficit irrigation, deeper rooting and increased root-to-shoot ratios promote water uptake, though occasionally at the expense of delayed reproductive growth or reduced fruit size [67,68]. Finally, CO_2_ supplementation has shown promise in stimulating photosynthesis and eventually growth under moderate heat stress (500–700 ppm) [69,70], though extreme temperatures may offset these gains by unsettling assimilate partitioning and reproductive sink strength [71,72,73].

Therefore, crop resilience under climate stress relies on physiological plasticity and genotype by environment interactions. Target traits include VPD sensitivity, stomatal control, photosystem repair, and antioxidant capacity [23]. In Mediterranean greenhouse systems, integrative practices combining genetic improvement, precision environment control, rootstock grafting, and DSS-based management are essential for preserving crop productivity [74].

Among the variety of physiological responses activated by climate-induced stressors in greenhouse cultivation, reproductive impairment, mainly during the flowering stage, serves as the most critical limitation to crop productivity (Table 2). High temperatures exceeding 32 °C, often in combination with elevated VPD, directly weaken pollen viability, anther dehiscence, and stigma receptivity, ultimately decreasing fruit set and yield in all three crops. Equally significant is the inhibition of photosynthesis, driven by thermal disruption of Rubisco activity, damage to photosystem II, and stomatal closure, limiting CO_2_ uptake. Oxidative stress, accompanied by the accumulation of ROS, further disrupts membrane integrity and cellular function under prolonged heat, drought, and radiation exposure. Moderate but still impactful responses include water stress-induced loss of turgor, elevated respiration rates leading to carbohydrate imbalance, and disrupted nutrient uptake and transport, particularly calcium-related disorders (e.g., BER). While adaptive responses such as altered root architecture and antioxidant activation provide some resilience, they are often insufficient under compounding stress. Overall, understanding the relative significance of these physiological disruptions is essential for prioritizing breeding targets and climate adaptation strategies in Mediterranean greenhouse systems.

**Table 2:** Classification of climate-induced physiological responses in greenhouse crops. BER, blossom-end rot; VPD, vapor pressure deficit.

Physiological Response	Relative Significance	Primary Stressors	Key References
Reproductive impairment (flowering, fruit set)	Very High	Heat, VPD	[75,76]
Photosynthetic inhibition	High	Heat, Radiation	[77,78]
Oxidative stress	High	Heat, Drought, Radiation	[62,79,80]
Water stress (turgor loss, transpiration)	Moderate to High	Heat, Drought, VPD	[79,81,82]
Respiration imbalance (e.g., carbohydrate depletion)	Moderate	Heat, Drought	[83,84]
Nutrient transport disruption (e.g., BER)	Moderate	Heat, Drought	[58,85]
Root morphology adaptation	Low to Moderate	Drought	[86]
Antioxidant response	Low to Moderate	Heat, Oxidative	[87,88]

Among the array of climate-induced stressors (Table 1), heat stress appears to be the most consistently detrimental across species and regions, especially when combined with high VPD (Table 3). In contrast, while drought and radiation stress are significant too, their impacts can be alleviated to a certain extent through precision irrigation, shading, and protective coverings. Elevated CO_2_, although potentially favorable, does not offset the negative consequences of extreme heat, when not managed alongside to other variables.

When comparing the three crops, cucumber generally exhibits the highest sensitivity to climate-induced stress, especially to thermal and VPD extremes, which affect floral development and fruit water status (Table 4). Tomato shows intermediate tolerance, with cultivar variability influencing resilience. Sweet pepper is relatively more tolerant in terms of reproductive heat stress thresholds and antioxidant responses, but remains vulnerable to water and nutrient imbalances. These differences underscore the importance of crop-specific management and breeding strategies tailored to future Mediterranean climate projections.

The impact of combined abiotic stressors, such as concurrent heat and drought or elevated VPD with high light intensity, represents a far greater threat to plant physiological functioning than single stress factors alone [118]. In real greenhouse conditions, these stresses rarely occur in isolation, and their interaction often leads to synergistically amplified damage. For instance, when high temperatures coincide with elevated VPD, stomatal closure limits CO_2_ uptake while transpiration-driven evaporative cooling is impaired, leading to leaf overheating and accelerated senescence. Similarly, drought combined with excessive radiation disorders photosynthetic electron flow and antioxidant enzyme balance, resulting in increased ROS accumulation and eventually oxidative damage. Notably, the combination of stresses not only impacts distinct physiological pathways but also overwhelms plant capacity for acclimation and recovery [119,120]. Therefore, assessing plant responses under multifactorial stress scenarios is instrumental for developing robust mitigation approaches and breeding programs that reflect real-world greenhouse environments in a changing Mediterranean climate.

### 3.2. Yield and Quality

As documented in a series of representative case studies (Supplementary Table S2; see also Supplementary Table S1), and compiled in Table 5, climate change-induced stressors exert critical effects on both yield and quality of greenhouse-grown vegetables in the Mediterranean basin. Regional case studies illustrate how elevated temperatures, radiation stress, water scarcity, and their interactions disturb physiological processes, degrade fruit quality, and diminish postharvest longevity. These challenges have encouraged a diverse collection of adaptation resources, including technological, agronomic, and varietal innovations tailored to regional climatic patterns.

Reproductive development is particularly vulnerable to heat stress, with day temperatures exceeding 32–35 °C and elevated night temperatures degrading pollen viability, ovule fertilization, and fruit initiation in tomato and sweet pepper [119,120,121,122]. Due to its floral biology, cucumber similarly suffers from weakened synchrony between male and female flower development or reduced ovary viability in parthenocarpic varieties [123,124]. These reproductive failures lead to reduced fruit set, lower yields, and increased prevalence of physiological disorders (e.g., BER, fruit cracking, and sunscald), particularly under high VPD and limited water availability.

Fruit quality parameters are highly sensitive to preharvest stress conditions [125]. In tomato, excessive heat during ripening diminishes lycopene synthesis, translated into poor coloration and inferior flavor quality due to reduced sugar and acid accumulation [126]. Sweet pepper and cucumber also exhibit deteriorations in nutritional value, antioxidant content, or increased bitterness when subjected to suboptimal cultivation environments [127,128]. Furthermore, fruit water content and turgor are negatively affected, impacting texture and storage stability [129]. Notably, these preharvest stresses carry over into the postharvest phase too, accelerating respiration and water loss, as well as increasing susceptibility to pathogen attack, and thereby shortening shelf life.

To mitigate these effects, Mediterranean countries have adopted a variety of climate adaptation measures, as featured in the selected case studies (Supplementary Table S2; see also Supplementary Table S1). Soilless cultivation systems, such as hydroponics and substrate-based setups, are widely employed in water-scarce regions (e.g., Spain and Turkey), allowing high WUE and precision fertigation control. In Greece and Cyprus, passive and semi-passive greenhouse designs, incorporating natural ventilation, thermal screens, whitewash shading, and reflective covers, are progressively employed to lower internal temperatures and improve reproductive performance.

In Spain and other regions, the integration of DSS allows for real-time optimization of irrigation, fertigation, and microclimate control based on sensor feedback and weather forecasting, participating in resource savings and yield stabilization. In Italy, Libya and Spain, photovoltaic (PV)-integrated greenhouse roofs operate the dual role of generating renewable energy and reducing internal heat load, thereby lowering cooling energy demand by up to 40% [130].

Moreover, genetic adaptation plays an imperative role in safeguarding stable productivity [131,132]. In Turkey and Egypt, heat- and drought-tolerant cultivars, often developed through regional breeding programs or molecular selection, have led to substantial improvements in fruit yield and quality under climate stress conditions. In Spain and Morocco, biological control and Integrated Pest Management (IPM) programs have radically reduced pesticide use, alleviating pest and disease pressures induced by rising temperatures.

Overall, Table 5 and the associated Supplementary Table S2 collectively emphasize the multifactorial basis of climate-induced impacts on yield and quality, as well as the importance of integrative and locally adapted mitigation measures. These policies lay the foundation for building resilient greenhouse production systems capable of enduring future climate volatility across the Mediterranean basin.

Among the various abiotic stressors, heat stress, particularly when combined with VPD, exerts the most critical impact on both yield and quality (Table 5). It disturbs reproductive processes, speeds physiological disorders, and exaggerates oxidative stress. Water scarcity inflicts considerable challenges too, particularly during fruit development stages. Radiation stress further intensifies these effects by elevating canopy temperatures and stimulating sunscald. Elevated CO_2_ levels sustain some mitigation through enhanced photosynthesis, although they are insufficient to offset heat or drought stress in isolation. When multiple stressors overlap, such as heat combined with drought and intense solar radiation, the influence on crop productivity and marketability becomes even more pressing, demanding robust integrated adaptation strategies.

**Table 5:** Relative impact of climate stressors on yield and quality of greenhouse crops. BER, blossom-end rot; RH, relative air humidity; T, temperature; VPD, vapor pressure deficit; WUE, water-use efficiency.

Climate Stress Factor	Impact on Yield	Impact on Quality	Key References
Heat stress (high T + high VPD)	Very High—disrupts reproduction, increases abortion rate	Very High—promotes BER, fruit cracking, color and flavor loss	[38,85]
Water stress (deficit irrigation, low RH)	High—limits cell expansion and nutrient transport	High—reduces firmness, size, and nutritional content	[48,133]
Radiation stress (excess solar radiation)	Moderate—exacerbates temperature stress and photodamage	Moderate—increases sunscald and oxidative damage	[11,134]
Elevated CO_2_	Variable—may increase WUE, but not offset heat effects	Variable—may boost photosynthesis but degrade sugar/acid balance	[135,136]
Combined stress (heat + drought + light)	Critical—synergistic effects severely reduce productivity	Critical—results in misshapen, low-quality fruits	[137,138]

Among the major climate change-related stressors (Table 1), heat stress appears the most critical to both yield and quality, predominantly due to its intense effects on reproductive development, physiological disorders, and postharvest performance (Table 3). Water stress, while substantial too, is often partially alleviated through irrigation technologies and substrate management. Radiation stress, although less commonly the single limiting factor, aggravates the adverse effects of heat and drought by increasing leaf temperatures and eliciting oxidative stress. Elevated CO_2_ levels, while being a potential mitigation pathway, do not consistently counteract the negative impacts of other stressors.

When comparing the three crops, cucumber emerges as the most sensitive, particularly due to its high water demand and vulnerability to reproductive disruption under heat and VPD stress (Table 4). Tomato shows intermediate resilience, benefiting from a broader range of adaptable genotypes and relatively stable reproductive performance. Comparatively, sweet pepper is more tolerant, especially in terms of antioxidant capacity and lower occurrence of fruit deformation, though it remains sensitive to calcium-related disorders and postharvest water loss.

Importantly, the combined occurrence of stressors, such as heat and drought, or radiation and VPD, reflects real-world greenhouse situations more accurately than single factors [137,138,139,140]. The synergistic effects of multiple stresses often enlarge the physiological damage, exceeding the sum of separate impacts [76,141]. For instance, concurrent heat and water stress can simultaneously reduce pollen viability, increase ROS production, and impair fruit expansion, eventually resulting in severe yield penalties. This fortifies the requirement for integrated, multi-factorial approaches in both research and cultivation setups aimed at developing climate-resilient greenhouse systems.

### 3.3. Pest and Disease Pressure

As highlighted in an array of representative case studies (Supplementary Table S3), and assembled in Table 6, climate change exerts an escalating influence on pest and disease pressures in greenhouse vegetable production systems across the Mediterranean zone. These impacts are displayed in shifting pest phenology, amplified viral outbreaks, impaired plant immunity, and a decay in the efficacy of traditional and biological control strategies. Regional case studies emphasize the pressing need for adaptive and integrated pest management tailored to new climate realities.

Warmer winters across the region enhance the overwintering success of major greenhouse pests such as *Bemisia tabaci* (whiteflies), *Frankliniella occidentalis* (thrips), and *Tuta absoluta* (tomato leafminer) [142,143,144]. This results in earlier seasonal emergence, prolonged activity periods, and a superior number of pest generations per year [142,145,146]. These factors collectively uplift crop exposure and enhance the frequency and intensity of infestations.

Vector-borne viruses have also become more prevalent and more severe. Viral diseases, such as tomato spotted wilt virus (TSWV), cucumber mosaic virus (CMV), and tomato yellow leaf curl virus (TYLCV) are spreading more aggressively due to amplified populations and reproductive rates of insect vectors under elevated temperatures [147,148,149]. Warmer temperatures not only improve vector fitness but also speed virus transmission, aggravating yield losses in heat-sensitive cultivation periods [147,148].

Moreover, climate-induced abiotic stress, particularly heat and water scarcity, destabilizes plant defenses, setting crops more vulnerable to opportunistic pathogens [150,151]. For example, *Botrytis cinerea* and *Fusarium* spp. thrive under combined heat and moisture stress in tomato and pepper, while erratic humidity levels promote *Pseudoperonospora cubensis* (downy mildew) outbreaks in cucumber [152,153]. Importantly, the fluctuating thermal and RH conditions that represent many Mediterranean greenhouse environments diminish the efficacy of fungicides too, further challenging typical disease control protocols [102].

Biological control, a key pillar of IPM, is progressively confronted by these shifting conditions. For instance, beneficial predators and parasitoids, such as *Phytoseiulus persimilis*, *Orius* spp. and *Encarsia formosa*, show downgraded efficacy and establishment rates under high temperatures or acute VPD conditions [154,155,156]. This underscores their suppressive character and can result in superior reliance on chemical pesticides, accelerating the risk of pest resistance.

Representative case studies (Supplementary Table S3) further clarify how different Mediterranean countries are currently acting. In Spain, *Tuta absoluta* is managed through mass trapping and enriched IPM practices. Israel and Cyprus have implemented UV-blocking films, insect-proof screens, and resistant cultivars to restrain TYLCV, while Egypt and Turkey employ reflective mulches and vector exclusion techniques to limit CMV. In Cyprus and Morocco, early planting and netting are used to decrease viral incidence during high-temperature windows. Responses to fungal and bacterial pathogens include soil solarization (Morocco, Tunisia, and Malta) [157,158,159], sulfur fumigation (France) [160], and improved ventilation strategies (Italy) [161].

An emerging tendency is the deployment of climate-integrated DSS, which incorporate real-time weather and pest data to optimize intervention timing [162,163]. These tools, implemented in Spain and Israel, assist in decreasing overapplication of agrochemicals while improving control precision. Climate-resilient crop breeding is gaining importance too, with several countries employing cultivars with combined resistance to heat and disease pressure [131,164].

Among the various abiotic stressors (Table 1), elevated temperature, especially when displayed as prolonged heatwaves or sustained high daytime and nighttime temperatures, appears as the most substantial factor influencing pest and disease dynamics in greenhouse horticulture (Table 6). It directly speeds insect metabolic rates, reduces pest life cycles, and increases virus transmission, thereby intensifying both the prevalence and severity of biotic stressors [147,148,165]. In contrast, RH fluctuations incline to impact a narrower range of pathogens, mainly favoring fungal and bacterial diseases under high moisture levels [152,166]. However, the effects of RH are typically secondary to thermal stress. VPD, while a strong marker of transpirational stress in plants, also plays a role in determining host–pathogen interactions by altering plant tissue susceptibility [167]. Elevated CO_2_ concentrations show complex and frequently ambiguous effects. Although elevated CO_2_ level can promote vegetative growth, it may simultaneously modify plant defense chemistry, potentially enhancing vulnerability to specific pests [168]. Overall, thermal stress exerts the most significant and universal influence on pest and disease dynamics (Table 3 and Table 6). Therefore, managing temperature extremes is the highest priority for mitigating climate-driven biotic threats.

**Table 6:** Critical abiotic stress factors affecting crop yield and quality in relation to pest and disease pressure. RH, relative air humidity; T, temperature; VPD, vapor pressure deficit.

Abiotic Stress Factor	Impact on Crop Yield and Quality	Relative Importance	Key References
Elevated T	Enhances pest development, increases virus vector efficiency	High	[147]
High RH	Promotes fungal and bacterial disease outbreaks	Moderate	[91]
Erratic RH	Triggers downy mildew and foliar diseases in cucumber	Moderate	[153]
Elevated VPD	Reduces efficacy of biocontrol agents and beneficial insects	High	[155]
Heat and Water Stress	Weakens plant immunity, facilitates *Botrytis* and *Fusarium* infections	High	[150,152]
Fluctuating T and RH	Limits fungicide efficacy and curative potential	High	[152,154]

When comparing the three crops, tomato appears to be the most susceptible to biotic stresses, predominantly to viral diseases such as TYLCV and TSWV, as well as to fungal pathogens like *Botrytis cinerea* (Table 4). Cucumber is moderately sensitive, especially to RH-driven diseases like downy mildew and to CMV. Sweet pepper exhibits relatively greater resilience, owing to thicker cuticles and stronger antioxidant responses, but still faces significant risks from thrips and powdery mildew under combined heat and water stress. This variability highlights the importance of crop-specific biotic stress mitigation strategies.

Critically, combined abiotic stressors magnify plant vulnerability to pests and diseases [119,168]. Heat stress, for instance, not only speeds pest reproduction but also undermines plant defense signaling pathways [147,148,168]. Simultaneous exposure to high VPD and water deficits reduces stomatal defense functions and nutrient mobility, aggravating disease incidence and pest outbreaks [23,169]. This junction of stress factors generates feedback loops which render traditional single-factor control methods insufficient, requiring integrative pest and disease management frameworks that account for multi-stress environments.

## 4. Water and Energy Demands

As underscored in the cluster of representative case studies (Supplementary Table S4), and composed in Table 7, water scarcity and elevated energy demand are among the most pressing challenges in Mediterranean greenhouse horticulture. Climate change has intensified both concerns, escalating ET rates, exhausting freshwater resources, and rising the energy required for sustaining optimal growing conditions.

Greenhouse vegetable crops are highly sensitive to microclimatic shifts, principally elevated temperatures and VPD, which radically boost ET [76]. Thereby, irrigation demands advance considerably, mainly during the summer season, struggling already limited water supplies in arid regions, such as southern Spain, Greece and Turkey [170,171,172]. Declining rainfall patterns and overexploitation of groundwater additionally complicate this concern [173]. To tackle these pressures, growers are progressively employing precision irrigation techniques supported by tensiometers, capacitance sensors, and DSS to optimize water application (timing and volume).

Alternative water sources (e.g., desalinated seawater, treated wastewater, and brackish groundwater) are being explored despite infrastructure demands and related (operational) costs [174,175]. In Tunisia, solar-powered desalination units are being integrated into irrigation systems, while Algeria and Morocco use subsurface irrigation and reverse osmosis desalination supported by solar power to handle water scarcity in low-tech greenhouses [176].

Soilless cultivation systems (e.g., hydroponics, aeroponics) offer high WUE, especially in closed-loop configurations, which minimize leaching and allow nutrient recycling [177,178]. In southern Greece (Crete) and Turkey, these systems are increasingly combined with automated fertigation platforms to conserve resources without compromising yield potential.

Simultaneously, the energy footprint of greenhouse operations has considerably risen. Cooling systems (e.g., evaporative pads, high-pressure foggers and mechanical ventilation) dominate energy consumption during peak heat periods, frequently accounting for more than 50% of total energy use [179]. In response, many regions are transitioning toward integrated renewable energy solutions. Greenhouses in Sicily and Puglia (Italy), as well as Izmir (Turkey) are deploying hybrid energy systems, including PV panels, thermal storage units, and solar microgrids to power cooling and irrigation infrastructure. These practices not only decrease greenhouse gas emissions, but also moderate reliance on unstable or expensive grid electricity.

Smart integration of water and energy management is a dominant tendency [180,181]. In Spain, whitewashed plastic coverings and aluminized shade nets lower internal heat load, while drip fertigation systems, controlled by soil moisture feedback, optimize water and nutrient use. In Sardinia (Italy), vertical drainage systems collect runoff for reuse, enhancing both WUE and nutrient cycling. Artificial intelligence (AI)-driven platforms like iGreenhouse use ET forecasts and solar radiation data to adjust fertigation and climate control dynamically.

Throughout the Mediterranean, greenhouse producers are converging on common strategies: the co-optimization of water and energy inputs, increased reliance on real-time sensor data, and integration of renewable technologies into day-to-day operations. While methodologies differ depending on regional climate severity and infrastructure availability, the causal drive stays the same, namely ensuring sustainable and resilient greenhouse production in the face of accelerating climate change.

In Mediterranean greenhouses, water and energy demands are predominantly driven by a combination of climatic stressors (Table 1), with high temperature and elevated VPD emerging as the most decisive (Table 3 and Table 7). These stressors speed ET, escalating irrigation needs and eliciting considerable cooling energy consumption [76]. Although water scarcity is a dominant matter, it is commonly manageable through efficient irrigation systems and alternative sourcing [182]. However, when high temperatures coincide with water limitations, especially under intense radiation, the combined effect considerably accelerates both resource use and operational challenges. This synergistic stress scenario emphasizes the importance of integrated adaptation strategies, which address water and energy simultaneously. Greenhouses are argued to embrace automation, renewable energy integration, and precision irrigation technologies to remain viable under future climate conditions.

**Table 7:** Critical Abiotic Stress Factors for Water and Energy Demands in Mediterranean Greenhouses. ET, Evapotranspiration; T, temperature; VPD, vapor pressure deficit. Arrows indicate direction of projected change (↑ increase, ↓ decrease).

Abiotic Stress Factor	Impact on Water Demand	Impact on Energy Demand	Relative Significance	Key References
High T	↑ ET; ↑ irrigation frequency and volume	↑ Cooling energy consumption	High	[183,184]
Elevated VPD	↑ ET; ↑ irrigation frequency and volume	↑ Ventilation and fogging requirements	High	[76,141]
Water Scarcity	↓ Water availability; necessitates efficiency and reuse	↑ Pumping energy for water sourcing	Moderate to High	[185]
Radiation Intensity	↑ Leaf T; ↑ water demand for cooling	↑ Shading and ventilation requirements	Moderate	[44,186]
Combined Heat and Water Stress	Synergistic increase in irrigation needs	Multiplicative effect on cooling and irrigation energy use	Very High	[118,187]

Among the three crops under analysis, cucumber is the most sensitive to combined water and heat stress (Table 4). Its rapid growth and high water content make it particularly vulnerable to dehydration and yield reduction under insufficient irrigation or high VPD. Tomato exhibits moderate sensitivity, with notable cultivar differences in drought tolerance and cooling needs. Sweet pepper generally shows greater resilience to water limitation and temperature variation, particularly when grown under appropriate rootstocks or using protective shading techniques. These species-specific traits must guide the prioritization of adaptation strategies.

It is critical to note that water and energy challenges rarely occur in isolation [187]. In real-world greenhouse environments, crops are simultaneously exposed to high temperature, VPD, radiation intensity, and limited water availability [184]. This combined stress scenario amplifies system vulnerabilities and increases the complexity of resource management. Greenhouses must therefore transition toward holistic solutions, incorporating automation, climate modeling, and renewable energy, to achieve sustainable and resilient vegetable production under a changing Mediterranean climate.

## 5. Adaptation Strategies

### 5.1. Greenhouse Structural Innovations

As featured in the assortment of representative case studies (Supplementary Table S5; see also Supplementary Tables S1–S3), an extensive array of structural innovations has been employed in greenhouse vegetable production across the Mediterranean zone to alleviate the expanding challenges of climate change. Structural modifications have arisen as a foundational adaptation strategy, augmenting climate regulation, resource-use efficiency, and overall crop performance under progressively stressful environmental conditions.

Among the most important interventions is the optimization of natural and forced ventilation systems [188,189]. Automated roof and side vents, ridge ventilators, and side curtains ease dynamic air exchange, lowering internal heat buildup through high solar radiation periods. In Almería (Spain), the integration of ridge and side ventilation systems resulted in a 5 °C temperature drop and an 18% increase in tomato yield, highlighting their thermal and agronomic benefits [190]. These developments are particularly essential under low external wind speeds, where internal convective cooling is the major channel of heat dissipation.

Shading technologies are additionally extensively utilized to balance excessive solar radiation [11,186,191]. Retractable shading screens and photo-selective nets allow both thermal protection and spectral filtering, by excluding undesirable wavelengths [e.g., near-infrared (NIR) radiation] while allowing transmission of photosynthetically active radiation (PAR) [192,193]. Studies from Crete (Greece) indicated that such shading decreased BER and promoted lycopene accumulation in tomato, while in Spain, reflective nets advanced sunscald resistance and ripening uniformity in sweet pepper [11].

Advanced greenhouse covering materials also increase thermal performance [194,195]. Thermally reflective films, double polyethylene layers with air gaps, and infrared (IR)-absorbing coatings diminish internal heat loads, while refining insulation [196,197,198]. In Italy, the use of insulating clear covers reduced seasonal energy consumption by 30%, and IR-absorbing films lessened leaf temperature and promoted chlorophyll biosynthesis in cucumber [199,200].

Architectural aspects, including structural geometry and orientation, further promote overall crop performance [201]. Higher ridge heights encourage vertical air movement and thermal stratification, while north–south orientations enable uniform light distribution. In the Mediterranean region, east–west greenhouse orientations assisted the reduction in energy requirements [187]. Sidewall insulation, using reflective or low-emissivity materials, diminishes heat exchange with the environment, assisting to sustain stable internal temperatures and moderate crop stress.

Integrated structural systems (e.g., external roll-up screens, misting devices combined with ventilation, and internal buffer zones) provide synergistic climate control [202,203]. NIR reflective greenhouse films have been displayed to lower the internal temperature and accordingly diminish the incidence of physiological disorders such as BER and fruit cracking in tomato [204]. In Mediterranean greenhouses, recent innovations (e.g., improved insulation materials, double claddings, and climate-smart structural designs) are gradually embraced to improve energy efficiency and crop resilience [205].

Collectively, these structural adaptations not only tackle climate-induced stressors (e.g., excessive temperature, radiation, and RH imbalance), but also operate as critical components of a holistic greenhouse management system. When supported with smart irrigation, climate-responsive varietal selection, and precision environmental monitoring, structural innovations fortify the resilience and sustainability of Mediterranean greenhouse horticulture under future climate volatility.

Among the various abiotic stressors addressed by structural greenhouse modifications (Table 1), heat stress and radiation excess are the most decisive due to their direct and immediate effects on crop physiology and yield (Table 3 and Table 8). Ventilation systems and shading technologies have appeared to be the most effective interventions, particularly for alleviating thermal buildup and solar overload during the summer period. While RH imbalances and cold stress are relevant too, predominantly in seasons of high evaporative demand or low night temperatures, their impacts are more variable and can be counterbalanced through misting, insulation, or thermal screens. Wind stress has localized importance, necessitating structural reinforcement mainly in exposed coastal or upland areas. Structural innovations, when carefully tailored to prevailing stress challenges, permit climate-resilient crop production by maintaining favorable microclimatic conditions in protected cultivation.

Crop-specific responses to structural innovations are apparent (Table 4). Tomato, being highly sensitive to high temperatures during flowering, shows noticeable yield and quality improvements under advanced ventilation and shading regimes. Cucumber, with its high leaf area index (LAI) and excessive transpiration rates, benefits most from enhanced insulation and misting strategies that regulate RH and consequently prevent leaf dehydration. Sweet pepper exhibits broader tolerance, though it displays evident gains in uniformity and reduced physiological disorders (e.g., sunscald) when cultivated under improved shading and sidewall insulation. Therefore, while all three crops benefit from structural upgrades, the extent and nature of response are noticeably species dependent.

Structural innovations are particularly effective in alleviating the joint effects of combined climate stressors, such as high temperature co-occurring with high radiation and VPD, which typically appear in the summer period [9]. By modulating internal microclimate through multiple physical parameters (light, heat, RH), structures such as ventilated ridge designs, reflective cladding, and hybrid insulation systems safeguard the crop from simultaneous stress exposure. These innovations facilitate maintaining a homeostasis across physiological systems, diminishing cascading damage from joint abiotic pressures and stabilizing yield potential under real-world cultivation scenarios.

### 5.2. Water Management

As noted in the compilation of representative case studies (Supplementary Table S6; see also Supplementary Tables S1, S2 and S5), a wide display of innovative water management strategies has been deployed in greenhouse vegetable production across the Mediterranean zone to mitigate the escalating challenges of climate change. As water shortage becomes more critical owing to declining precipitation patterns, elevated ET rates, and intensifying salinity, efficient and adaptive irrigation practices are fundamental to supporting vegetable production in protected cultivation (Table 9).

Precision irrigation, predominantly drip systems integrated with tensiometers, soil moisture sensors, and automated fertigation units, appears as the basis of water-saving interventions [212,213]. These technologies allow real-time, site-specific water delivery based on crop development stages, root-zone conditions, and environmental features, consequently augmenting WUE and diminishing nutrient leaching [180,210]. In Almería (Spain), this practice advanced WUE by over 30% without limiting yield, while comparable findings were obtained in Turkey and France via sensor-guided irrigation control.

DSS further improve irrigation scheduling by integrating weather forecasts, ET models, and soil moisture dynamics [214,215]. Their utilization in Greece, Portugal and Spain indicates how such means empower adaptive, demand-driven responses to climate variability. When combined with wireless sensor networks and cloud-based analytics, DSS platforms enable remote monitoring and automated adjustments, upgrading responsiveness and minimizing both water and energy inputs [216].

Hydroponic and closed-loop systems embody high-efficiency alternatives, which recycle drainage water while preserving nutrient stability [177,194]. These systems, increasingly deployed in Israel and Greece, are associated with significant decreases in both water and fertilizer usage but involve close management of salinity and pathogen risk. Water reuse strategies are also gaining importance across the region [217]. In Egypt, Lebanon and Algeria, recirculation of treated drain water by means of UV, ozone, or biofiltration ensures microbiological quality and nutrient retention, diminishing environmental discharge and sustaining circular resource use.

Alternative water sources are imperative in coastal or drought-prone regions [175]. Desalinated seawater is employed in Tunisia, Algeria and Turkey, while rainwater harvesting systems are integrated into greenhouse configurations in Greece. Although desalination is energy-demanding, its coupling with solar PV systems, as in Turkish greenhouses, facilitates to counterbalance environmental and financial costs.

Holistic schemes, which combine irrigation with climate control measures, offer synergistic gains [47,218]. Regulated deficit irrigation (RDI) linked with fogging or shading, as realized in Spain and Palestine, facilitates to alleviate physiological stress while conserving water. Such joint procedures have been denoted to restrain BER incidence and uphold fruit firmness, even under restricted water availability [118,218]

Collectively, these case studies designate that climate-resilient greenhouse water management relies not only on technology but also on localized adaptation. The successful deployment of precision irrigation, water reuse, alternative sources, and integrated climate strategies converges on contextual suitability, crop-specific needs and long-term sustainability planning. As the Mediterranean horticultural sector confronts escalating hydrological challenges, these scalable solutions shape the agenda of resource-efficient and productive greenhouse crop production.

In Mediterranean greenhouse systems, water management needs to cope with manifold abiotic stressors (Table 1), among which high temperature and water scarcity surface as the most critical (Table 9). Elevated temperatures considerably amplify ET rates, amplifying irrigation demand and frequently dictating integration with cooling technologies (e.g., fogging or shading). Water shortage, instead, directly constrains resource availability, impelling for widespread adoption of precision irrigation, water reuse, and alternative sourcing such as desalination [210,213]. Salinity stress, commonly resulting from poor-quality or recycled water, necessitates cautious management via blending or advanced filtration [219]. Elevated VPD, an outcome of increasing temperature and decreasing RH, further inflates plant water loss and complicates irrigation scheduling. Erratic rainfall and radiation stress further insert layers of randomness and energy burden, respectively. These stressors jointly require highly responsive, integrated water management methodologies tailored to local climatic and crop-specific requirements.

**Table 9:** Abiotic stress factors affecting water management. ET, Evapotranspiration; T, temperature; VPD, vapor pressure deficit.

Stress Factor	Relative Significance	Impact on Water Management	Key References
High T	Very High	Increases ET, requires cooling integration, accelerates water loss	[117,206]
Water Scarcity	Very High	Limits water availability, necessitates reuse and alternative sources	[217,220]
Salinity Stress	High	Challenges water quality, requires filtration/blending	[208,209]
Elevated VPD	High	Increases transpirational demand, complicates irrigation scheduling	[76,210]
Erratic Rainfall	Moderate	Reduces reliability of natural water sources, necessitates storage	[172,211]
Radiation Stress	Moderate	Increases canopy T, indirect effect on water demand	[11,186]

From a stress-type perspective (Table 1), raised ET rates owing to intensifying temperatures and VPD exercise the greatest pressure on water resources, pressing both quantitative and qualitative water stress (Table 3). However, water quality decline, predominantly salinity buildup from recycled or alternative water sources, progressively jeopardizes irrigation sustainability [207]. While erratic rainfall participates in uncertainty [221], the long-term increase in ET and salinity most directly shapes water-use decisions and system design.

When comparing the three crops, cucumber emerges as the most sensitive to water-related stress owing to its shallow root system and elevated transpiration, leading to rapid turgor loss and fruit quality deterioration under deficit irrigation (Table 4). Tomato displays intermediate tolerance, benefiting from some osmotic adjustment and moderate WUE. Sweet pepper is relatively more resilient, particularly under RDI regimes, since it sustains fruit set and quality over a wider range of moisture conditions. Nevertheless, even sweet pepper becomes vulnerable when water scarcity is coupled with acute heat or salinity stress [127,128].

Combined stress, rather than single-factor restrictions, expresses real-world greenhouse challenges [222]. Water scarcity sporadically appears in isolation, since it is commonly combined with heat stress, salinity, and nutrient imbalances. Such multifactorial stress strengthens physiological pressure, diminishing both water uptake and transport efficiency. Combined RDI and heat, for instance, impair fruit expansion, activate calcium-related disorders (e.g., BER), and accelerate senescence [129]. Therefore, successful water management in Mediterranean greenhouses gradually relies on integrative, multi-stress mitigation techniques merging irrigation technology, climate buffering, and crop-specific strategies.

### 5.3. Variety Selection and Agronomic Practices

As displayed in the synthesis of representative case studies (Supplementary Table S7; see also Supplementary Tables S1 and S2), and integrated in Table 10, region-specific efforts have been implemented under the scope of advancing crop resilience through climate-adaptive cultivar selection, breeding, and agronomic practices. Since vegetable crops are highly sensitive to abiotic stressors, principally heat, water unavailability and oxidative stress (Table 2), climate-smart variety development is vital for maintaining yield and quality under increasingly challenging environmental conditions.

One of the most essential responses to climate pressure encompasses the selection and utilization of heat-tolerant cultivars [223,224]. These are increasingly deployed across the Mediterranean zone, as the ‘Arawak’ tomato hybrid in Spain and the drought-resilient tomato lines in Tunisia. Such cultivars regularly retain superior pollen viability, durable flowering performance, robust root systems, and upgraded antioxidant defenses, attributes which collectively participate in reproductive success and fruit quality maintenance during high temperature periods [14,225]. Case studies from Egypt further underscore the significance of antioxidant-rich cultivars which sustain marketability across heatwaves.

Molecular breeding techniques [e.g., marker-assisted selection (MAS), genomic selection, and CRISPR/Cas-based editing] have noticeably advanced the development of stress-resilient cultivars [226,227]. Italy and France have employed MAS to enhance WUE and thermal tolerance in tomato and sweet pepper, while Israel and Spain have engaged advanced genomic tools to improve flowering stability and oxidative stress resilience. These methodologies complement standard phenotypic selection under field stress conditions, allowing a quicker, more targeted route to trait incorporation [228,229,230].

Grafting continues to be a widely adopted mitigation solution in response to abiotic stress [225,231]. In Greece, Turkey and Spain, the deployment of stress-tolerant rootstocks for tomato and pepper has advanced nutrient and water uptake efficiency, while minimizing susceptibility to BER and other physiological disorders linked to thermal and hydric stress.

Agronomic adjustments, such as shifting transplanting dates or selecting fast-cycling cultivars, allow strategic avoidance of peak stress periods [232]. Shifting tomato planting from late to early spring or utilizing early-harvest cucumber hybrids, as performed in Egypt and Turkey, can facilitate the alleviation of yield loss during terminal heatwaves. These practices improve resilience without demanding extensive infrastructural changes.

A noteworthy evolving tendency is the incorporation of omics technologies (e.g., transcriptomics, proteomics, and metabolomics) for trait discovery and precision [233]. Turkey and Italy are expanding this approach to develop genotypes with grander WUE, oxidative stress tolerance, and reproductive stability under elevated temperatures. These system-level insights assist the breeding of next-generation climate-smart cultivars.

Eventually, the examples recorded across Mediterranean regions (Supplementary Table S7; see also Supplementary Tables S1 and S2) underpin the importance of integrating genetic improvement, physiological optimization, and responsive agronomy. The combined employment of climate-resilient genotypes and adaptive practices (e.g., protected cropping, precision irrigation and smart climate control) offers a robust strategy to maintain productivity and quality in greenhouse vegetable production systems under the on-going climate change.

Variety selection exerts a critical influence on greenhouse crop resilience, principally in the context of climate-induced abiotic stresses [234]. Among the diverse stressors, high temperature is the most decisive element impacting varietal performance, largely owing to its direct effect on flowering, pollen viability and fruit set (Table 10). Accordingly, breeding for thermotolerance is the major priority in most Mediterranean programs. Water scarcity is highly important too, impelling attempts to improve WUE and root system resilience. Oxidative stress, while frequently a downstream effect of heat and drought, instructs the selection of cultivars with enhanced antioxidant systems. Salinity stress is moderately important, principally in coastal regions or areas employing alternative irrigation sources [235,236] and is progressively tackled via rootstock selection [225]. Radiation stress is commonly alleviated via structural solutions rather than varietal features, setting it as a lower breeding priority. This classification emphasizes the requirement for integrated breeding approaches, which prioritize heat and drought resilience, but incorporate secondary traits for comprehensive climate adaptation.

**Table 10:** Abiotic stress prioritization for variety selection in greenhouse crops. T, temperature; WUE, water-use efficiency.

Abiotic Stress Factor	Significance for Variety Selection	Key References
High T (Heat Stress)	Very High—Directly affects reproductive success and yield stability; top priority for thermotolerant breeding	[38,97]
Water Scarcity (Drought Stress)	High—Critical during flowering and fruiting; influences WUE and root architecture traits	[220,237]
Oxidative Stress	Moderate—Often a secondary effect of heat/drought; relevant for antioxidant-related breeding targets	[62,195]
Salinity Stress	Moderate—Important in coastal/irrigated areas; included in some advanced rootstock and varietal programs	[208,209]
Radiation Stress	Low—Limited direct breeding focus; mitigated via structural and shading adaptations	[11,186]

Among the various stress factors (Table 1), high temperature stress surfaces as the most restrictive during reproductive development, directly affecting fruit set and quality (Table 3). Drought stress, though equally critical, frequently tolerates greater scope for alleviation through irrigation management and rootstock selection. Oxidative stress is commonly a secondary consequence of heat or water restriction but still exerts a key role in disturbing membrane integrity and photosynthetic efficiency. Therefore, cultivar selection endeavors incline to prioritize thermotolerance and reproductive stability, tailed closely by WUE and antioxidant capacity.

While sweet pepper displays prominent physiological resilience under moderate stress, principally in terms of WUE, antioxidant capacity and postharvest integrity, it presents high sensitivity at the varietal level (Table 4). This is mainly related to the restricted availability of genotypes with stable reproductive performance under combined heat and water deficits, as well as its pronounced susceptibility to calcium-related disorders (e.g., BER). Tomato demonstrates intermediate tolerance, with several cultivars acquiring adaptive features including reproductive thermotolerance and flexible phenological responses. Cucumber, although resilient in water uptake and vegetative development under moderate stress, is sensitive to the accumulation of bitter compounds (cucurbitacins) under intense heat and drought conditions, which unfavorably influences marketability [238]. These species-specific vulnerabilities underscore the requirement for targeted breeding programs tailored to the projected climatic constraints of the Mediterranean zone.

In actual cultivation scenarios, multiple stressors, particularly heat and drought, often coincide [118], designating combined stress tolerance as a principal breeding objective. Combined stresses tend to aggravate physiological disruptions more severely than individual ones, demanding multifactorial screening routines during cultivar development [139]. This complexity underlines the importance of integrative strategies which select for resilience under concurrent stress conditions, combining traits (e.g., stable photosynthesis, robust antioxidant systems, and strong reproductive performance) to safeguard consistent productivity under real-world Mediterranean greenhouse settings.

### 5.4. Renewable Energy Use

As revealed in the overview of representative case studies (Supplementary Table S8), climate adaptation strategies in Mediterranean greenhouse horticulture encompass energy efficiency, renewable integration, and cross-sectoral resilience. As climate change exaggerates, the necessity to decrease energy dependency, alleviate escalating operational costs, and sustain productivity has directed greenhouse energy optimization to the center of sustainable horticultural practices.

The core of this evolution is the incorporation of renewable energy technologies [239,240]. Solar PV panels are extensively implemented to provide electricity for critical systems including fans, pumps, and lighting [241]. Complementary solar thermal systems and geothermal installations supply heating and cooling, downgrading dependence on fossil fuels [242]. Hybrid systems combining PV with thermal energy storage permit for more balanced energy supply through diurnal and seasonal cycles [243]. These technologies not only alleviate greenhouse gas emissions but also safeguard farmers against unstable electricity prices and grid instabilities.

At the same time, passive energy-saving solutions are deployed to advance internal climate regulation [239]. Thermal screens composed of aluminized or multilayered materials moderate heat loss at night and during low radiation periods [187]. The incorporation of phase change materials (PCMs) into greenhouse structures empowers the capture and controlled release of surplus thermal energy, stabilizing internal temperatures without enlarging energy consumption [244]. Moreover, structural enhancements (e.g., reflective roof covers, whitewashing, and insulation layers) assist in the decrease in solar heat load, while sustaining optimal light levels for crop photosynthesis [183,198].

Advanced environmental control systems have emerged vital for optimizing energy use [245]. These systems pull real-time data from sensors and weather forecasts to dynamically handle ventilation, shading, irrigation, and temperature [246]. AI-based controllers, such as those integrated in platforms like Hort@ and iGreenhouse, proactively adjust energy-consuming processes to enhance efficiency while safeguarding optimal cultivation conditions. Energy modeling and simulation tools, including computational fluid dynamics (CFD), are employed to design optimum ventilation and insulation configurations [247], while life cycle assessments (LCA) assess the environmental influence of different technology combinations [248].

Energy storage systems, including batteries connected to solar arrays, improve energy self-sufficiency by delivering power during periods of low generation [245]. Intelligent load management additionally enlarges renewable use by associating high-demand activities (e.g., cooling or fertigation) with peak energy production periods [249].

Notably, these energy strategies are combined with climate-smart water management, genetic innovation, and digital agriculture platforms [250,251]. Precision irrigation systems, often combined with tensiometers, capacitance sensors, and weather-driven DSS, diminish water and energy waste by supplying tailored irrigation based on real-time conditions [187]. Soilless systems [e.g., hydroponics and nutrient film techniques (NFT)] improve water and nutrient use efficiency, while decreasing energy costs linked with excessive pumping or pathogen control [252].

On the crop side, heat- and drought-tolerant cultivars (e.g., ‘SV8591’ tomato or *Solanum habrochaites*-grafted hybrids] fortify reproductive success and resource efficiency under stress [223,253]. Agronomic adaptations, encompassing modified sowing times and fast-cycling varieties, facilitate to evade peak climatic extremes, upgrading synchronization with favorable environmental windows [254]. Other strategies involve evaporative cooling, dynamic shading, insect-proof netting, and chromatic screens, which decrease internal temperatures and constrain pest access without reliance on chemical inputs [9,255]. Postharvest quality is further preserved through cold chain systems with RH regulation, decreasing spoilage and dehydration during transport in warmer conditions [79]. Finally, farmer training, participatory research, and technology demonstration programs guarantee practical implementation. These capacity-building endeavors are critical to scaling up the adoption of innovative technologies among dissimilar Mediterranean production systems.

Among the various stressors aggregating energy use in Mediterranean greenhouses (Table 1), high air temperature and increased solar radiation intensity are identified as the primary drivers (Table 11; see also Table 3). These stressors considerably increase cooling needs, enlarge internal heat load, and aggravate energy consumption, principally in the summer months. Elevated VPD further indirectly contributes by intensifying transpiration rates, which raises the requirement for active cooling systems to sustain optimal microclimate. Instead, factors such as low RH and water scarcity exert a more indirect role in energy demand, by impacting irrigation needs and pumping requirements. Consequently, strategies intended for renewable energy integration and passive thermal regulation ought to principally tackle high temperature and radiation loads, since these pose the most significant challenges to energy efficiency and sustainability in protected cultivation.

In greenhouse horticulture, the relative importance of distinct renewable energy strategies differs according to the stressor being tackled and the production context. Solar PV systems stand for the most widely adopted technology owing to their modularity, relatively low cost, and compatibility with a range of greenhouse operations, including ventilation, fertigation, and supplemental lighting [240]. Instead, geothermal and hybrid PV-thermal systems, while highly efficient, necessitate substantial upfront investment and technical infrastructure [242,245], setting them more appropriate for large-scale or institutional greenhouses. Passive systems (e.g., thermal screens and PCMs) are principally significant in buffering thermal extremes and preserving energy during nocturnal cooling or cold spells [256,257,258]. These approaches are crucial under combined climate stresses, where energy needs peak erratically, and they deliver favorable baseline efficiency, which complements active renewable systems.

Among the three crop species considered, tomato tends to profit most from renewable energy deployment owing to its longer cultivation cycles and greater sensitivity to suboptimal climate regulation during critical reproductive stages (Table 4). Retaining stable temperature and RH through smart energy systems is indispensable for fruit set, lycopene synthesis, and diminishing disorders such as BER [54,245]. Equally, cucumber, with its high transpiration rates and sensitivity to VPD, benefits considerably from upgraded cooling and RH control, predominantly under high solar radiation conditions. Sweet pepper, although moderately sensitive, displays greater resilience in certain stages but still benefits from energy-enabled climate buffering to decrease physiological disorders and sunscald. Accordingly, species-specific benefits from renewable energy integration expose discrepancies in phenology, physiology, and stress thresholds.

Notably, in real-world Mediterranean conditions, renewable energy systems ought to deal with combined stresses, namely elevated temperature, radiation overload, and irregular water availability [245]. These stressors not only lift energy demand but also amplify the risk of crop failure in the absence of responsive microclimate regulation. Renewable energy integration permits for the simultaneous operation of shading systems, evaporative cooling, and automated ventilation without intensifying operational costs. The synergistic employment of active and passive energy-saving systems safeguards resilience against multifactorial stress combinations, which are increasingly common owing to climate variability. Eventually, climate-smart energy strategies qualify greenhouse systems to sustain productivity, moderate emissions, and boost economic sustainability under the complex pressures of climate change.

### 5.5. Comparative Cost–Benefit Overview

While Section 5.1, Section 5.2, Section 5.3, Section 5.4 described the technical aspects of individual adaptation measures, their feasibility in practice also depends on economic efficiency [259,260]. Cultivators in the Mediterranean zone must balance initial investment costs, operational and maintenance expenses, and the long-term benefits of improved yield stability, water- and energy-use efficiency, and reduced climate risk [32,163,261]. To provide a clearer comparison, Table 12 summarizes the indicative cost–benefit profile of the main adaptation strategies, based on representative case studies from the region [199,262,263].

This comparative analysis shows that low- to medium-cost options, such as optimized irrigation technologies and stress-resistant cultivars, provide the most immediate and broadly applicable solutions [262,263,264]. By contrast, capital-intensive measures, such as renewable energy integration and structural retrofitting, although highly effective in reducing long-term vulnerability, are often constrained by high initial costs and slow return on investment [265,266]. These findings highlight the importance of supportive policy instruments and further applied research to reduce adoption barriers and improve long-term cost efficiency [267,268]. The specific roles of policy frameworks and research priorities in enabling these transitions are discussed in Section 8.

It should be noted that Table 12 also includes DSS and digital tools, even though these are analyzed in detail in the following section. Their inclusion here allows for a more complete cost–benefit comparison alongside the core structural, agronomic, and energy-related strategies (Section 5.1, Section 5.2, Section 5.3, Section 5.4) [163,269]. Section 6, therefore, expands specifically on DSS applications, validation efforts, and adoption barriers in Mediterranean greenhouse systems.

**Table 12:** Comparative cost–benefit overview of major adaptation strategies in Mediterranean greenhouse horticulture. DSS, Decision Support System; PV, Photovoltaic; WUE, water-use efficiency.

Strategy	Initial Investment Cost	Operational/Maintenance Cost	Performance Benefits	Overall Feasibility in Mediterranean Context	Key References
Greenhouse structural improvements (e.g., shading screens, thermal insulation, natural ventilation upgrades)	Medium–High	Low–Medium	Lowers heat load, reduces energy demand, stabilizes yields under heat stress	Moderate to high, often dependent on subsidies or cooperative investment	[270,271,272]
Water-saving irrigation (e.g., drip irrigation, fertigation, DSS-based scheduling)	Medium	Low	+30–40% WUE, +10–20% yield stability under drought	High, widely adopted and cost-effective	[181,273,274]
Stress-resistant cultivars/rootstocks	Low	Low	Improves tolerance to heat and drought, enhances yield stability; moderate yield gains	High feasibility; adoption increasing across the region	[275,276,277]
Renewable energy integration (e.g., PV panels, geothermal heating/cooling)	High	Low–Medium	Substantial reduction in fossil energy use, long-term cost savings, lower CO_2_ footprint	Feasible mainly with policy incentives or external financing	[271,278]

## 6. Decision Support and Digital Tools

The integration of digital technologies into greenhouse vegetable production has scaled essential for adjusting to the escalating struggle of climate change [117,279]. Mediterranean greenhouses must compact with a hazardous combination of thermal stress, water insufficiency, pest dynamics, and energy constraints. DSS are developing as central tools for tackling these multidimensional confronts with precision and prescience.

DSS platforms merge real-time data from in situ sensors, local weather stations, and satellite imaging with predictive models to deliver cultivators with actionable advice [117]. These platforms carry decisions on irrigation scheduling, nutrient management, pest control, ventilation, and energy usage [163,177,214,280]. The capability to foresee and alleviate both biotic and abiotic stressors drastically promotes resilience and resource efficiency [281,282]. Latest DSS platforms [e.g., Hort@ (Spain), iGreenhouse (Italy), NUTRISENSE (Greece) and AgriSens (Greece)] are combining cloud computing, Internet of Things (IoT) infrastructure, and AI-based learning algorithms. These technologies empower the shift from reactive to predictive cultivation. For instance, thermal imagery and Normalized Difference Vegetation Index (NDVI) data obtained from drones can be fed into crop growth models to predict yield and discriminate stress hotspots before symptoms are visible.

An essential consideration in evaluating greenhouse-focused DSS and crop/climate simulation models is the robustness of their validation. Across the studies reviewed, validation datasets typically range from small-scale greenhouse trials (n = 20–50 observations) to multi-year, multi-site evaluations involving more than 200 observations, depending on the tool and context. Such validation efforts are critical to ensure statistical reliability and to confirm that these tools can be transferred across the diverse agro-climatic conditions of the Mediterranean region [99,283,284].

Remote sensing tools and edge computing improve the competence of DSS by allowing high-resolution, real-time analytics [282]. Edge devices are able to locally handle sensor data (e.g., soil moisture, canopy temperature, CO_2_ levels, nutrient concentration) to decrease invisibility and advance autonomy in decision-making [285,286]. Linked with mobile app interfaces, these tools permit continuous communication between systems and farmers, even in distant or resource-limited sites [287]. An exceptionally promising novelty is the employment of digital twin models, which are virtual representations of greenhouse environments simulating crop responses under different climate conditions [288]. These models assist to assess the cost-effectiveness of suggested adaptation strategies and simplify experimental design without disturbing actual production cycles.

Notwithstanding their potential, the extensive adoption of DSS in Mediterranean countries is yet restrained. Barriers involve the necessity for standardized data formats, affordable sensor packages, robust internet connectivity, and tailored training programs to safeguard user competence. Collective initiatives implicating research institutions, agritech companies, and policy stakeholders are essential to tackle these breaches and endorse technology transference to small- and medium-scale producers. By merging the predictive ability of AI with granular field data, DSS and digital tools embody a keystone of climate-smart greenhouse horticulture [289]. As climate change persists to unsettle traditional practices, the implementation of these platforms will develop progressively principal to resilient, efficient, and sustainable protected cultivation systems in the Mediterranean zone [290].

Digital technologies and DSS are quickly modernizing Mediterranean greenhouse management by qualifying more precise, adaptive, and resource-efficient cultivation approaches. Table 13 portrays a range of digital platforms and tools tailored to deal with the multifaceted and dynamic encounters caused by climate change, such as irregular weather patterns, water shortage, and amplified energy demands. These tools differ in scope, from integrated greenhouse control systems which automate irrigation and climate regulation, to crop modeling platforms which simulate plant responses under diverse stress scenarios. For instance, platforms like iGreenhouse, NUTRISENSE, and AgriSens integrate environmental sensors and AI-driven analytics to provide real-time instructions on temperature, RH, irrigation, nutrient and salinity control. By administering multisource data, covering in situ sensors, remote sensing inputs, and meteorological forecasts, these tools improve the capacity of cultivators to react proactively to changing conditions [32,163,261,285].

In a related way, crop-specific DSS platforms such as Hort@ deliver tailored advising for Mediterranean vegetable crops, incorporating phenological models with localized agronomic schedules. These systems are specifically constructive for optimizing sowing, harvesting, and input application agendas, hence associating production cycles with periods of minimum climatic stress [291]. Table 13 also contains tools which assist broader planning and policy integration (e.g., such as ClimaView and SmartFarmNet), which are employed for regional-scale monitoring and the design of adaptive strategies. These platforms ease cooperation among cultivators, researchers, and policymakers by envisioning tendencies in resource use, crop stress, and greenhouse performance.

A crucial attribute among these tools is their accessibility. Many are cloud-based, mobile-compatible, and organized with multilingual interfaces to endorse widespread adoption among Mediterranean growers. Training packages and technical support are frequently embedded within the platforms to expand usability and warrant long-term engagement.

Nevertheless, validation remains a key bottleneck: many platforms still rely on limited datasets, and future work should prioritize expanding validation sample sizes and standardizing benchmarking protocols to strengthen robustness and scalability [284,292]. In short, Table 13 emphasizes the essential role of digital agriculture in encouraging climate-resilient greenhouse production. By reinforcing real-time decision-making, predictive planning, and resource optimization, these digital tools epitomize crucial elements of sustainable horticultural systems in the Mediterranean region. Their additional development and integration, predominantly when combined with grower feedback and policy support, can meaningfully increase the region’s adaptive capability.

**Table 13:** Digital tools and Decision Support Systems (DSS) in climate-smart greenhouse horticulture ranked by country. AI, Artificial Intelligence; IoT, Internet of Things; NUE, nitrogen-use efficiency; WUE, water-use efficiency.

Tool/Platform	Country	Crop	Functionality	Key Features	Outcomes	Key References
iGreenhouse	Italy	Tomato	Climate & Irrigation Control	AI, IoT, Cloud-based	Improved water and energy efficiency	[273,293]
NUTRISENSE	Greece	All	Crop & Cultivation system- specific DSS	Mobile-compatible, cloud based	Improved WUE and NUE, yield increase and custom-made nutrition	[177,283,294]
AgriSens	Greece	Pepper	Sensor Integration & DSS	Mobile-compatible, edge computing	Real-time adaptation to stress	[295]
Hort@	Spain	Tomato	Crop-specific DSS	Phenological models, local calendars	Optimized timing, yield stability	[296,297]
ClimaView	France	All	Regional Monitoring	Remote sensing, dashboards	Supports planning & resilience	[298]
SmartFarmNet	Cyprus	Cucumber	Greenhouse management	Multi-sensor, predictive analytics	Climate response modeling	[299]

Between the diverse stressors (Table 1), thermal stress and water scarcity are the most dominant drivers behind the adoption of DSS platforms (Table 3). Elevated temperatures disturb crop development and amplify ET rates, necessitating precise microclimate control and irrigation adjustments, situations where DSS can deliver real-time solutions [214]. Water availability, mainly in southern Mediterranean regions, necessitates fine-tuned irrigation to match plant requirements and resource conservation. Instead, although energy usage and pest dynamics are imperative too, their impression on DSS adoption is frequently inferior and more context-specific. Consequently, DSS systems are most extensively implemented where temperature and water-related stress intersect and entail constant monitoring and intervention.

Regarding crop-specific gains, tomato cultivation emerges to benefit the most from DSS integration, mainly owing to its high sensitivity to abiotic stress during flowering and fruit set, as well as its economic magnitude in the Mediterranean basin (Table 4). Cucumber profits considerably too, mainly from tools which optimize water and temperature control through its rapid growth cycle. Sweet pepper, while responsive to DSS-based climate and fertigation control, displays relatively greater resilience to temperature fluctuations and consequently exhibits slightly lower reliance on high-frequency decision inputs. Nevertheless, all three crops reveal appreciable enhancements in yield and resource efficiency when supported by advanced DSS tools.

The joint stress state, which comprises synchronized exposure to high temperatures, water deficits, pest pressures, and energy limitations, is progressively the standard in Mediterranean greenhouse systems [184]. DSS platforms are distinctively set to tackle these overlapping stressors, since they can combine multisource data and deliver adaptive algorithms to upgrade responses across systems [163]. The holistic class of these tools renders them indispensable for overseeing trade-offs between competing resource needs and for alleviating cascading stress effects which may not be manageable via isolated mediations. As previously described, DSS represent a strategic integrative solution for coping the compound, interconnected nature of climate stress in protected cultivation.

## 7. Regional Case Studies

This section organizes important adaptation strategies and regional case studies, underscoring how cultivators are refashioning greenhouse practices, adopting digital tools, and choosing resilient cultivars to maintain production under altering climatic conditions. As condensed in Table 14, the cases expose a series of technologies and approaches tailored to national frameworks.

Spain: In Almería, cultivators have established one of the most technologically advanced greenhouse clusters in Europe, spanning more than 30,000 hectares [300]. Confronting rising temperature extremes and erratic rainfall patterns, producers have realized high-tech solutions involving multi-layered reflective screens, automated climate control, and recirculating hydroponic systems [301]. DSS platforms like Hort@ are extensively operated for optimizing fertigation and scheduling irrigation based on real-time ET models. Research stations cooperate closely with cultivators to test and disseminate innovations [302]. Pest control persists a substantial challenge owing to prolonged activity periods of whiteflies and *Tuta absoluta*.

Greece: In the island of Crete, the majority of greenhouses are unheated, plastic-covered structures prone to seasonal thermal extremes [303]. Cultivators are progressively transitioning cultivation schedules to evade peak summer heat, and have commenced adopting grafted plants employing rootstocks tolerant to drought and salinity. Tensiometer-based irrigation and closed-loop fertigation systems are aiding to upgrade WUE [304]. DSS platforms like NUTRISENSE and AgriSens are being progressively adopted, mainly among cooperatives, offering precise crop nutrition and weather-integrated alerts for irrigation and disease risk [177,283]. Extension services and EU-funded projects support the upscaling of these technologies [283,305].

Turkey: In coastal regions such as Antalya and Mersin, vegetable production is principal to both national supply and exports [306,307]. Escalating temperatures and elevated VPDs have compromised traditional ventilation systems. Cultivators are operating high-pressure fogging and insect-proof ventilation structures to upgrade climate control. PV-integrated greenhouses are in pilot stages, intending to counterbalance energy costs. Turkish-developed DSS platforms are currently integrating AI-based phenology models and mobile decision tools which aid to optimize pesticide application windows and fertigation scheduling [308,309]. Government support via subsidies and training has enhanced technology uptake.

Italy: In the Emilia-Romagna region, high-tech greenhouses are equipped with geothermal heating systems, reflective roof coatings, and energy-efficient light-emitting diodes (LEDs) [310]. Climate prediction models embedded within iGreenhouse are employed for proactive climate control [163]. High-throughput phenotyping and data-driven monitoring are typical in research and commercial sites. Growers operate mobile dashboards to remotely manage irrigation and ventilation [311]. DSS platforms integrate phenology, market demand, and weather forecasts to fine-tune harvest timing [311]. The strong connection between academic research centers and producer organizations eases rapid innovation.

France: Greenhouse vegetable producers in Provence are utilizing integrated systems which link climate-adapted cultivars with smart irrigation infrastructure [312]. Soil moisture sensors are combined with ET forecasts to manage automated drip systems. DSS tools provide daily irrigation advice adjusted for local microclimate and crop phenology. Varieties bred for heat resilience and enhanced shelf life are progressively adopted [313]. Regional policy programs co-finance technology upgrades and offer subsidies for sensors, software, and training programs.

Egypt: The transition to controlled-environment horticulture is sustained by national programs focusing on solar-powered greenhouses, mainly in areas with extreme temperatures and water shortage [314]. Hydroponic systems, employing NFT and perlite substrates, are being scaled in peri-urban areas. Weather-linked DSS platforms and mobile advisories are fostered to small-scale producers, improving planning and input efficiency. Partnerships with international donors strengthen capacity building, while local start-ups are developing solar-battery integration modules to sustain nighttime climate control [315].

Tunisia: Cultivators are adopting climate-resilient practices such as utilizing grafted pepper and tomato lines, while biological pest control (e.g., *Trichogramma* spp.) is progressively substituting chemical treatments [316,317,318]. Mobile apps which deliver weather alerts and fertilization instructions are increasing in reach among farmer cooperatives. Low-cost tensiometers and solar-powered drip systems are progressively accessible. Pilot projects with non-governmental organizations and universities assess DSS functionality in smallholder settings. Public awareness campaigns encourage resilience knowledge and user-friendly digital interfaces.

Morocco: Agadir region is a renowned greenhouse hub, with more than 20,000 hectares of predominantly Canarian-type greenhouses, which specializes in tomato and pepper production [317,319]. Greenhouses in this region are often unheated and rely on favorable local climate, but recent heatwaves and water scarcity issues have posed significant challenges. To address these challenges, there is a growing transition toward mid- and high-tech greenhouse systems through public–private partnerships. These upgraded systems incorporate efficient water management via recirculation and monitoring, integrated climate control, and data-driven crop management. National training programs and agricultural extension workshops are introducing DSS tools which utilize sensor feedback and satellite imagery, enabling cultivators to manage microclimates more effectively [320]. Complementary strategies include enhancements in postharvest cold chains and regional trials of heat-tolerant tomato cultivars. These efforts are part of “Morocco’s Green Generation Plan”, which emphasizes digital agriculture as a central development pillar.

Across these regional cases, several common adaptation patterns can be identified. In response to water scarcity, drip irrigation, fertigation, and closed-loop systems, often supported by DSS-assisted scheduling, have become widespread throughout the Mediterranean basin. High energy costs are increasingly addressed through PV integration, improved insulation, and cooperative energy-sharing schemes. Cultivar resilience, particularly grafted plants and heat-tolerant tomato and pepper lines, emerges as another cross-cutting strategy. DSS platforms, whether national (e.g., NUTRISENSE, AgriSens, Hort@) or international, are progressively supporting irrigation, fertigation, and climate control decisions.

Beyond technical innovations, socio-economic constraints strongly shape adoption. High initial investment costs, fragmented farm structures, and limited access to credit frequently restrict the uptake of capital-intensive measures such as renewable energy systems or advanced climate control. Policy incentives (e.g., subsidies for solar installations in Spain, EU-funded irrigation upgrades in Greece, cooperative financing models in Italy, or Morocco’s Green Generation Plan) are essential in lowering these barriers. Summarizing these cross-country experiences highlights that while national contexts differ, growers across the basin face similar drivers and rely on a recurring set of adaptation strategies. These shared lessons provide a foundation for designing transferable policies and practices across the Mediterranean region. These regional experiences not only highlight technical innovations but also underline the socio-economic and policy enablers required for their wider adoption, which are further discussed in Section 8.

**Table 14:** Regional case studies on greenhouse climate adaptation in the Mediterranean basin. AI, Artificial Intelligence; DSS, Decision Support System; ET, Evapotranspiration; NFT, nutrient film technique; PV, Photovoltaic.

Country	Adaptation Strategies	Technologies and Tools	Key References
Spain	High-tech greenhouses, hydroponics, climate control, DSS	Reflective screens, Hort@ DSS	[74,300,301]
Greece	Seasonal shifts, grafted plants, precision irrigation, DSS	Tensiometers, NUTRISENSE, AgriSens DSS	[177,303,304]
Turkey	Fogging, insect-proofing, PV integration, DSS	Turkish DSS platforms, solar panels	[307,308,309]
Italy	Geothermal heating, high-throughput phenotyping, DSS	iGreenhouse, AI analytics	[163,310]
France	Smart irrigation, climate-adapted cultivars	Soil sensors, ET-based scheduling	[312,313]
Egypt	Solar greenhouses, hydroponics, national adaptation schemes	Solar-PV, NFT	[314,315]
Tunisia	Grafted heat-tolerant crops, mobile DSS, low-cost pest management	Mobile apps, organic biocontrols	[316,317,318]
Morocco	Transition to Mid-Tech and Hi-Tech structures, DSS training	DSS tools, training platforms	[319,320]

## 8. Policy Framework and Research Priorities

The formulation of coherent policies and targeted research agendas is crucial for the sustainability and climate resilience of greenhouse vegetable crops. Building on Section 5.5, this section examines key policy instruments and research priorities supporting adaptation in Mediterranean greenhouse systems [321]. As climate challenges intensify (Table 1), coordinated national and transnational frameworks must foster innovation, reduce vulnerability, and ensure equitable access to adaptation technologies for producers of all scales. Regional evidence shows that water scarcity, high energy costs, and cultivar resilience are common drivers, highlighting the need for harmonized, basin-wide policy instruments.

Financial incentives are essential to help greenhouse operators, particularly small and medium-sized enterprises (SMEs), transition to climate-resilient practices. Subsidies for renewable energy, climate control, and precision irrigation, along with simplified permitting and tax relief, can accelerate this shift. European Union (EU) instruments such as the Green Deal, Common Agricultural Policy (CAP), and Farm to Fork Strategy offer opportunities for greenhouse-specific adaptation goals but often lack regional focus. Expanded support for Mediterranean horticulture, including neighboring countries (e.g., Turkey and Tunisia), is needed to reflect local vulnerabilities [26,251].

Developing stress-resilient cultivars remains a key research priority, combining genomic tools, phenotyping, and participatory breeding to enhance tolerance to heat, drought, and pests. Research should also advance low-carbon, low-input greenhouse systems integrating passive regulation, efficient lighting, and recyclable materials [239,240]. DSS require continuous refinement and localization through open-access, multilingual platforms linking sensor data, weather forecasts, and crop models. Ensuring user-friendly design is vital for widespread adoption [322].

International cooperation through platforms such as Partnership for Research and Innovation in the Mediterranean Area (PRIMA) and Horizon Europe accelerates technology diffusion and harmonization of standards. Regional trials, farmer field schools, and climate-smart hubs promote participatory innovation and bridge research–practice gaps. Long-term monitoring programs are vital for assessing adaptation progress and guiding improvements. Integrating local knowledge and farmer experience enhances relevance and social acceptance, ensuring the co-production of knowledge essential for Mediterranean greenhouse resilience.

Table 15 summarizes the strategic dimensions of climate adaptation in Mediterranean greenhouse production, linking policy tools, financial mechanisms, and research directions aimed at enhancing resilience, sustainability, and profitability. It shows how transnational frameworks (e.g., the Green Deal, CAP, and Farm to Fork Strategy) underpin greenhouse adaptation but must be contextualized for Mediterranean high-value crops exposed to combined thermal, hydric, and pest pressures.

National subsidy schemes for renewable energy and smart irrigation incentivize farm-level change, while simplified permitting, tax relief, and technical support reduce financial and bureaucratic barriers. Ensuring equitable access, especially for SMEs that dominate Mediterranean horticulture, remains crucial. Subsidies for solar energy, climate control, and precision irrigation directly reduce emissions, improve efficiency, and protect crops from extremes.

Table 15 outlines investment in localized and accessible DSS (e.g., multilingual, mobile platforms) that provide practical tools for adaptive crop management. It further describes advances in biotechnological breeding (e.g., genomics, CRISPR, high-throughput phenotyping) combined with participatory methods ensuring that farmer needs shape varietal development. The shift toward low-input greenhouse systems, which integrate passive ventilation, energy-efficient lighting, and biodegradable materials, supports both adaptation and mitigation goals, particularly under Mediterranean water and energy constraints [323,324]. Regional initiatives such as PRIMA and Horizon Europe foster cross-border learning and policy coherence, while incorporating local ecological knowledge increases the contextual relevance and acceptance of innovations.

The current body of research faces several limitations. Climate projections carry inherent uncertainty from model and data variability [325,326], while greenhouse simulation models in DSS or digital twins simplify plant–environment interactions [327,328], reducing reliability under multiple stresses [329,330]. Geographic concentration of studies also limits representativeness [331]. Future work should pursue coordinated, multi-site, multi-year trials and model inter-comparisons to enhance transferability [332,333]. Table 15 thus outlines a policy–research roadmap, emphasizing that technological progress must align with supportive governance, fair financing, and adaptive research. Synthesizing national experiences, this review identifies shared lessons for transferable, region-wide adaptation, vital for a sustainable, climate-resilient Mediterranean greenhouse sector.

**Table 15:** Policy instruments and research priorities for climate-resilient greenhouse horticulture in the Mediterranean basin. CAP, Common Agricultural Policy; DSS, Decision Support System; EU, European Union; PRIMA, Partnership for Research and Innovation in the Mediterranean Area; SMEs, Small and Medium-sized Enterprises.

Thematic Area	Policy/Research Priority	Description	Key References
Financial Incentives	Subsidies for Renewable Energy and Climate Control Technologies	Support adoption of solar, geothermal, and precision irrigation systems in greenhouses, especially for SMEs	[334,335,336,337]
Regulatory Support	Streamlined Permits and Tax Relief	Facilitate permits for sustainable greenhouse structures and offer tax incentives for investments in environmental monitoring and automation	[334,337]
EU Framework Integration	Alignment with EU Green Deal, CAP, Farm to Fork	Adapt existing EU policies to specifically address Mediterranean greenhouse needs, including regional vulnerabilities	[338,339]
Breeding Innovation	Development of Stress-Tolerant Cultivars	Invest in genomic-assisted breeding, high-throughput phenotyping, and farmer-participatory approaches to develop heat-, drought-, and pest-tolerant varieties	[340,341,342]
Sustainable Systems	Low-Carbon, Low-Input Greenhouse Design	Promote passive climate control, recyclable materials, and energy-efficient lighting in greenhouse construction	[343,344,345]
Decision Support Tools	Localized, User-Friendly DSS Platforms	Develop multilingual, open-access platforms integrating sensors, weather data, and crop models for grower decision-making	[346,347]
Research Collaboration	Regional Networks and International Projects	Leverage PRIMA, Horizon Europe, and knowledge hubs for joint trials, farmer schools, and standard harmonization	[340,348,349]
Monitoring and Evaluation	Agro-Environmental Monitoring Programs	Establish long-term programs for assessing climate adaptation performance and guiding iterative improvements	[350,351,352,353]
Local Knowledge	Inclusion of Farmer Experience and Ecological Knowledge	Use participatory research to enhance relevance, acceptance, and efficacy of adaptation strategies	[345,354]

## 9. Conclusions

Greenhouse cultivation in the Mediterranean zone is at the forefront of climate change impacts, tackling escalating temperatures, erratic precipitation patterns, and raised evapotranspiration rates, and exaggerating pest and disease pressures. These stressors jeopardize not only crop productivity and quality, but also the economic viability of greenhouse enterprises. Among the set of climate-induced stressors, heat stress stands out as the dominant environmental limitation influencing greenhouse crop performance. In real-world production systems, however, these stressors seldom arise in isolation. Instead, they interrelate simultaneously and synergistically, frequently compounding their adverse effects on crop physiology, yield, and resource demands. Despite these challenges, Mediterranean greenhouse systems present significant opportunities for adaptive transformation. Controlled environment agriculture offers a platform for implementing climate-smart technologies which improve resilience and sustainability.

This review combined current scientific and applied knowledge on the crop physiological responses to climate stress, innovative greenhouse structures, and energy- and water-efficient practices. Special emphasis was set on tomato, cucumber, and sweet pepper cultivation, extracting insights from regional case studies and underscoring the magnitude of integrated adaptation strategies. Of the three crops under consideration, cucumber displays the greatest sensitivity to climate stressors, while sweet pepper expresses comparatively higher resilience under adverse conditions. Technological innovations (e.g., sensor-driven irrigation systems, energy-efficient climate controls, and digital decision support platforms) are by now displaying their ability to decrease vulnerability while upgrading productivity and input efficiency. Additionally, advances in crop breeding, involving stress-resilient cultivars and grafted combinations, pose promising genetic solutions to environmental extremes.

Nevertheless, the full potential of these adaptations can only be fulfilled through an enabling policy environment and collaborative research plan. Financial incentives for infrastructure modernization, support for renewable energy deployment, and capacity-building initiatives for cultivators are vital. Equally important is the endorsement of knowledge exchange across Mediterranean countries to speed up technology transfer and context-specific innovation. A paradigm shift is required, from fragmented, reactive measures to a holistic, proactive redesign of greenhouse systems which focus on climate resilience, ecological sustainability, and socioeconomic completeness. By associating technological progress with supportive governance and farmer empowerment, Mediterranean greenhouse production can function as a resilient pillar of food security and rural development under the on-going climate change.

To translate these insights into actionable pathways, several priority tasks can be outlined. In the short term, emphasis should be placed on coordinated multi-site validation of DSS platforms, benchmarking cost–benefit trade-offs of adaptation strategies, and participatory breeding trials for stress-resilient cultivars. In the medium term, efforts should focus on scaling renewable energy integration, establishing regional greenhouse monitoring networks, and embedding climate adaptation targets within national agricultural policies. These steps will align scientific advances with socio-economic feasibility and accelerate the transition toward climate-resilient greenhouse horticulture in the Mediterranean.

Overall, the findings of this review highlight that technological solutions must be embedded within supportive governance, equitable financing, and adaptive research systems. Synthesizing across national contexts, shared lessons can inform transferable policies and practices, making adaptation strategies more broadly applicable throughout the Mediterranean basin. This integrated approach is indispensable for realizing a sustainable and climate-resilient greenhouse horticulture sector across the region.

## Figures and Tables

**Table 3:** Key environmental factors per impact area. Each impact area corresponds to a thematic section in the manuscript, and the listed environmental factors represent the dominant climate stressors influencing that area. Further details and mechanistic explanations are provided within the respective sections of the text. The temperature (T)-related factors (heat stress, high T, and thermal extremes) are considered together, without implying that they are interchangeable or equally stressful to plants. High T typically corresponds to an increase in vapor pressure deficit (VPD), which is also recognized as a climate change-associated stressor. DSS, decision support systems; ET, Evapotranspiration.

Impact Area	Most Important Environmental Factor	Key References
Physiological Responses	Heat stress, especially combined with high VPD	[89]
Yield and Quality	Heat stress affecting reproductive development and postharvest performance	[90]
Pest and Disease Pressure	High T (elevates pest survival and virus transmission)	[91]
Water and Energy Demands	High T and elevated VPD	[92,93]
Structural Innovations	Thermal extremes (e.g., high solar radiation, VPD)	[94]
Water Management	ET increase due to high T and elevated VPD; salinity buildup	[76,95]
Variety Selection	High T during reproduction; oxidative stress	[96,97]
Renewable Energy Use	Thermal extremes affecting climate regulation	[98]
DSS	Heat and water limitations as key drivers of adoption	[99]

**Table 4:** Species-specific responses across key climate adaptation impact areas. Each impact area corresponds to a thematic section of the manuscript, where the species-specific tolerance and sensitivity patterns, as well as yield-related responses, are further analyzed and discussed. The temperature-related factors (heat stress, and high temperature) are considered together, without implying that they are interchangeable or equally stressful to plants. High temperature typically corresponds to an increase in vapor pressure deficit (VPD), which is also recognized as a climate change-associated stressor. BER, blossom-end rot; Ca, calcium; DSS, Decision Support System; RDI, regulated deficit irrigation; WUE, water-use efficiency.

Impact Area	Crop			Key References
	Tomato	Cucumber	Sweet Pepper	
Physiological Responses	Intermediate tolerance; cultivar-dependent resilience	Most sensitive; affected by heat/VPD, floral disruption	Most tolerant; good antioxidant response, heat tolerance	[38,100,101]
Yield and Quality	Intermediate resilience; stable reproductive performance	Most sensitive; high water demand and VPD sensitivity	Most tolerant; low fruit deformation, strong antioxidant capacity	[97,102,103]
Pest and Disease Pressure	Most susceptible; prone to viruses and fungi	Moderate sensitivity; humidity-driven pathogens (e.g., downy mildew)	Least susceptible; thicker cuticles, moderate pest risk	[104,105,106]
Water and Energy Demands	Moderate sensitivity; cultivar-dependent drought and cooling responses	Most sensitive to water/heat overlap; rapid dehydration	Most resilient; performs well with RDI and shading	[48,102,107]
Structural Innovations	High yield gain with advanced ventilation/shading	Benefits from misting and insulation for humidity retention	Broad tolerance; benefits from shading and insulation	[108,109,110]
Water Management	Moderate WUE; osmotic adjustment possible	Highly sensitive due to shallow roots and high transpiration	Most resilient; maintains fruit set under varied moisture	[48,111,112]
Variety Selection	Intermediate; some thermotolerant cultivars available	Resilient vegetatively; but bitterness under high stress	Most sensitive during flowering (BER, Ca transport issues)	[58,65,97]
Renewable Energy Use	Highly benefits from energy support during reproduction	Needs improved cooling/humidity due to high transpiration	Moderate benefit; resilient but aided by buffering systems	[113,114,115]
DSS	Gains most from DSS due to stress sensitivity during flowering	Gains from DSS for rapid growth and climate regulation	Least DSS-dependent; resilient but improves with DSS	[116,117]

**Table 8:** Structural modifications and their relation to abiotic stress factors. ET, Evapotranspiration; T, temperature; VPD, vapor pressure deficit.

Stress Factor	Relative Significance	Impact on Water Management	Key References
High T	Very High	Increases ET, requires cooling integration, accelerates water loss	[206]
Water Scarcity	Very High	Limits water availability, necessitates reuse and alternative sources	[185,207]
Salinity Stress	High	Challenges water quality, requires filtration/blending	[208,209]
Elevated VPD	High	Increases transpiration rate, complicates irrigation scheduling	[76,210]
Erratic Rainfall	Moderate	Reduces reliability of natural water sources, necessitates storage	[211]
Radiation Stress	Moderate	Increases canopy T, indirect effect on water demand	[11,198]

**Table 11:** Abiotic stress factors and their significance for renewable energy use in Mediterranean greenhouses. RH, relative air humidity; T, temperature; VPD, vapor pressure deficit. Arrows indicate direction of projected change (↑ increase).

Abiotic Stress Factor	Impact on Energy Use	Relative Significance	Key References
High air T	↑↑↑ Cooling energy demand, overheating risk	Very High	[184,188]
Increased solar radiation	↑↑ Internal heat load, shading and insulation needed	High	[186,198]
Elevated VPD	↑↑ Transpiration-driven cooling needs	Moderate to High	[76]
Low RH	↑ Pumping energy demand for irrigation	Moderate	[220]
Water scarcity	↑ Indirectly affects energy via irrigation systems	Moderate	[185,217]

## Data Availability

No data are provided in this review.

## Supplementary Materials

The following supporting information can be downloaded at: https://www.mdpi.com/article/10.3390/plants14213390/s1, Table S1: Climate-induced stress responses in greenhouse crops ranked by stress type and crop. BER, blossom-end rot; Ca, calcium; ET, evapotranspiration; g_s_, stomatal conductance; K, potassium; Na, sodium; NPK; nitrogen, phosphorus, potassium; PGPR, plant growth-promoting rhizobacteria; RDI, regulated deficit irrigation; ROS, reactive oxygen species; Si, silicon; T, temperature; UV, ultraviolet; VPD, vapor pressure deficit; WUE, water-use efficiency; Table S2: Climate-induced responses on yield and quality in greenhouse crops ranked by stress type and crop. BER, blossom-end rot; ET, Evapotranspiration; IWR, integrated water resources; RDI, regulated deficit irrigation; SFS, Screenhouse-Fogging System; Si, silicon; T, temperature; TSS, total soluble solids; UV, ultraviolet; VPD, vapor pressure deficit; Table S3: Climate-induced responses on pest and disease pressure in greenhouse crops ranked by crop and pest/disease threat. The temperature (T)-related factors (heat stress, and high T) are considered together, without implying that they are interchangeable or equally stressful to plants. High T typically corresponds to an increase in vapor pressure deficit (VPD), which is also recognized as a climate change-associated stressor. BYI, before yellowing initiates; CMV, cucumber mosaic virus; FOL, *Fusarium oxysporum f.* sp. lycopersici; FORL, *Fusarium oxysporum f.* sp. radicis-lycopersici; IPM, Integrated Pest Management; RH, relative air humidity; ToBRFV, Tomato brown rugose fruit virus; TSWV, tomato spotted wilt virus; TYLCV, tomato yellow leaf curl virus; Table S4: Estimated water and energy demands in greenhouse crops ranked by crop and country; Table S5: Structural innovations in greenhouse crops ranked by crop and country. The temperature (T)-related factors (heat stress, and high T) are considered together, without implying that they are interchangeable or equally stressful to plants. High T typically corresponds to an increase in vapor pressure deficit (VPD), which is also recognized as a climate change-associated stressor. BER, blossom-end rot; CMV, cucumber mosaic virus; DSSC, dye-sensitized solar cell; gs, stomatal conductance; IR, infrared; NIR, near-infrared; RH, relative air humidity; RWC, relative water content; UV, ultraviolet; WUE, water-use efficiency; Table S6: Water management innovations in greenhouse crops ranked by crop and country. The temperature (T)-related factors (heat stress, and high T) are considered together, without implying that they are interchangeable or equally stressful to plants. High T typically corresponds to an increase in vapor pressure deficit (VPD), which is also recognized as a climate change-associated stressor. AI, artificial intelligence; BER, blossom-end rot; DM, Dry Matter; DSS, Decision Support System; EC, electrical conductivity; ET, evapotranspiration; EU, European Union; gs, stomatal conductance; IoT, Internet of Things; LCA, life cycle assessment; MSW, municipal solid waste; OM, organic matter; PV, Photovoltaic; RDI, regulated deficit irrigation; TA, Total Antioxidants; TDR, time domain reflectometry; TI, thermal index; TSS, total soluble solids; UV, ultraviolet; VPD, Vapor Pressure Deficit; WUE, water-use efficiency; Table S7: Variety selection and agronomic practices for climate resilience in greenhouse crops ranked by crop and country. The temperature (T)-related factors (heat stress, and high T) are considered together, without implying that they are interchangeable or equally stressful to plants. High T typically corresponds to an increase in vapor pressure deficit (VPD), which is also recognized as a climate change-associated stressor. BER, blossom-end rot; ET, evapotranspiration; MAS, marker-assisted selection; PV, Photovoltaic; RDI, regulated deficit irrigation; Si, silicon; TSS, total soluble solids; WUE, water-use efficiency; Table S8: Adaptation strategies for climate resilience in greenhouse crops ranked by thematic area. AI, Artificial Intelligence; DSS, Decision Support System; IoT, Internet of Things; IPM, Integrated Pest Management; NFT, nutrient film technique; PCMs, Phase-Change Materials; PV, Photovoltaic; RH, relative air humidity; T, temperature; UV, ultraviolet; WUE, water-use efficiency. References [355,356,357,358,359,360,361,362,363,364,365,366,367,368,369,370,371,372,373,374,375,376,377,378,379,380,381,382,383,384,385,386,387,388,389,390,391,392,393,394,395,396,397,398,399,400,401,402,403,404,405,406,407,408,409,410,411,412,413,414,415,416,417,418,419,420,421,422,423,424,425,426,427,428,429,430,431,432,433,434,435,436,437,438,439,440,441,442,443,444,445,446] are cited in the supplementary materials.

## Abbreviations

The following abbreviations are used in this manuscript:

AI	Artificial Intelligence
BER	Blossom-end rot
CAP	Common Agricultural Policy
CFD	Computational Fluid Dynamics
CMV	Cucumber mosaic virus
CO_2_	Carbon dioxide
DSS	Decision Support System
ET	Evapotranspiration
EU	European Union
FAO	Food and Agriculture Organization
IR	Infrared
IoT	Internet of Things
IPCC	Intergovernmental Panel on Climate Change
IPM	Integrated Pest Management
LCA	Life Cycle Assessment
MAS	Marker-assisted selection
NDVI	Normalized Difference Vegetation Index
NFT	Nutrient film technique
NIR	Near-infrared
PAR	Photosynthetically Active Radiation
PCMs	Phase-Change Materials
RDI	Regulated deficit irrigation
RH	Relative air humidity
ROS	Reactive oxygen species
PRIMA	Partnership for Research and Innovation in the Mediterranean Area
PV	Photovoltaic
SMEs	Small and Medium-sized Enterprises
TSWV	Tomato spotted wilt virus
TYLCV UNEP	Tomato yellow leaf curl virus United Nations Environment Programme
UV	Ultraviolet
VPD	Vapor Pressure Deficit
WUE	Water-use efficiency
