# Supplementary material for: Climate Change Impacts on Greenhouse Horticulture in the Mediterranean Basin: Challenges and Adaptation Strategies
Source: Plants (Basel). 2025 Nov 5;14(21):3390. doi: 10.3390/plants14213390 (PMC12608269; doi:10.3390/plants14213390)
Supplement: Supplementary file 1 [file plants-14-03390-s001.zip › plants-3956429-supplementary.pdf]

Table S1. Climate-induced stress responses in greenhouse crops ranked by stress type and crop. BER, blossom-end rot; Ca, calcium; ET, evapotranspiration; g_s, stomatal conductance; K, potassium; Na, sodium; NPK; nitrogen, phosphorus, potassium; PGPR, plant growth-promoting rhizobacteria; RDI, regulated deficit irrigation; ROS, reactive oxygen species; Si, silicon; T, temperature; UV, ultraviolet; VPD, vapor pressure deficit; WUE, water-use efficiency.

Country	Crop	Stress Type	Physiological Response	Intervention	Reference
Egypt	Cucumber	Heat stress	↑ ROS, ↓ chlorophyll and photosynthesis efficiency, ↓ water regulation	Heat-resistant genotypes	[65]
Italy	Pepper	Heat stress	↓ g_s, ↓ chlorophyll content,↑ leaf T, ↓ photosynthesis rate	Use of shading nets (Aluminet and Chromatinet)	[186]
Spain	Pepper	Heat stress	Damage in the photosynthetic apparatus and sunscald	Grafting as an alternative to shading screens	[231]
Croatia	Tomato	Heat stress	Leaf chlorosis and wilting, ↓ root and shoot growth, membrane damage, ↓ photosynthesis	Genetic screening of tolerant genotypes, monitoring HSP and TF gene expression, hormonal regulation	[355]
Egypt	Tomato	Heat stress	↓Flowering and fruit set, ↓ chromosomal stability	Selection of heat-tolerant lines (G6, G2), breeding programs	[356]
Egypt	Tomato	Heat stress	↓ Growth and yield, altered antioxidant enzyme activity; genotype-dependent oxidative stress response	Selection of heat-tolerant cultivars based on biochemical and yield performance	[357]
France	Tomato	Heat stress	↓ Fruit set, altered flower morphology, growth inhibition, oxidative damage, ↓ chlorophyll content	Selection of heat-resilient cultivars, identification of key thermotolerance genes, molecular breeding	[358]
Turkey	Tomato	Heat stress	↓Fruit set and yield, ↓ photosynthesis, ↓ nutrient uptake, accelerated ripening with poor quality	Expanded fog systems, renewable energy, water-use efficiency, cooling/shading, heat-tolerant varieties, adjusted cropping seasons	[359]
Israel	Tomato	Heat stress	↓ Fruit set, ↓ seed production, ↓ yield	Heat-tolerant cultivars	[360]
Spain	Pepper	Heat stress	↓ Leaf area and relative growth rate, ↓ photosynthetic efficiency, ↓ chlorophyll content, ↓ fruit set and ↓reproductive success, ↑ ROS	Grafting onto heat-tolerant rootstocks (A6, A25, A57)	[361]
Spain	Tomato	Heat stress	Photosynthesis inhibition, ↑ROS, ↓ growth	Application of oligosaccharides as biostimulants	[362]
Egypt	Cucumber	Heat and salinity stress	↓ Photosynthesis ↑ oxidative stress; membrane damage osmotic imbalance,	Foliar application of nano-selenium, Si, or H_2O_2	[222]

			↓ growth and yield (↓ shoot/root length, fruit size)		
Egypt	Cucumber	Heat and salinity stress	↓Growth, ↓yield	Grafting	[225]
Greece	Cucumber	Heat and UV stress	↓ Photosynthesis	Shading nets	[11]
Cyprus	Cucumber	Heat and UV stress	↑ Transpiration rates and ↓ nutrient solution	Application of whitewash shading and fan-pad evaporative cooling systems	[183]
Cyprus	Tomato	Heat and VPD stress	↑ Leaf T, ↑ transpiration rates, ↓ WUE, ↓ stomatal regulation	Use of model with real-time leaf T data to optimize irrigation and improve microclimate control	[206]
Mediterranean region	Cucumber Tomato, Sweet Pepper	Drought	↓ Water uptake, ↓ photosynthesis, ↓ nutrient uptake and growth	Grafting onto drought-tolerant rootstocks	[253]
Tunisia	Cucumber	Drought	↓ Yield, ↓WUE, ↓growth and physiological performance	Subsurface drip irrigation	[363]
Greece	Sweet Pepper	Drought	↓ Yield, ↓ nutrient content, ↓ fruit quality (↓ size, Ca, firmness)	Tolerant local landraces (higher leaf K concentration and lower Na fruit concentration)	[364]
Spain	Sweet Pepper	Drought	↓ Fruit biomass and number, ↑BER, ↓ plant water status	Shading, RDI	[47]
Spain	Sweet Pepper	Drought	↓ Fruit production, ↑ unmarketable fruits, ↓ biomass and yield	Irrigation at 100% ET	[220]
Greece	Tomato	Drought	↓ Plant growth and yield, ↓ fruit quality (size, firmness, nutrient content)	Tomato hybrids bred for low-input conditions, PGPR	[365]
Turkey	Tomato	Drought	↓ g_s, ↓ leaf water potential	Grafting with drought-tolerant cultivars	[366]
Greece	Cucumber	Drought and salinity stress	↓ Growth, ↓ photosynthesis, biochemical imbalances, ↓ fruit quality	Si application	[367]
Turkey	Sweet Pepper	Drought and Salinity	↓ Water consumption, ↓ yield, ↓ vegetative growth	CO_2 + night cooling	[235]
Greece	Cucumber	Salinity stress	↓ Yield potential	Soil salinity modeling and management under climate change scenarios	[368]
Greece	Pepper	Salinity stress	↓ Yield, ↓ nutrient content, ↓ fruit quality (↓ size, Ca)	Tolerant local landraces	[364]
Turkey	Pepper	Salinity stress	↓ Growth and biomass, ↓chlorophyll content	Application of 2 mM Si and 100 μM nitric oxide at 7- and 14-d intervals	[369]
Israel	Pepper	Salinity stress	↓ Root mass, conductivity, ↓ transpiration, ↓ biomass production	Implementation of an artificial capillary barrier in the root zone	[370]

Spain	Tomato	Salinity stress	↓ Growth and yield, ↓ fruit quality	Balanced NPK fertilization, soil salinity monitoring,irrigation management	[209]
Greece	Tomato	Salinity stress	↓ Growth and yield, ↓ fruit quality	Tolerant local landraces, biostimulants	[371,372]
Spain	Cucumbe r	High/low UV	↑ Respiration	Schedule irrigation, optimizing water use and maintaining plant cooling	[373]

Table S2. Climate-induced responses on yield and quality in greenhouse crops ranked by stress type and crop. BER, blossom-end rot; ET, Evapotranspiration; IWR, integrated water resources; RDI, regulated deficit irrigation; SFS, Screenhouse-Fogging System; Si, silicon; T, temperature; TSS, total soluble solids; UV, ultraviolet; VPD, vapor pressure deficit.

Crop	Country/R egion	Stress Factor	Yield Impact	Quality Impact	Mitigation Strategy	Reference
Cucumb er	Egypt	Heat stress	↓ Yield	Smaller, wilting fruits, ↓chlorophyll	Use of heat-tolerant genotypes	[65]
Sweet pepper	Spain	Heat stress	↓ Yield	↓ Fruit set and quality	Grafting onto heat-tolerant rootstocks	[361]
Sweet pepper	Spain	Heat stress	↓ Fruit set and marketable yield	↓ Fruit quality and reproductive success	Grafting ‘Herminio F1’ onto heat-tolerant rootstock A57	[374]
Tomato	Mediterren ean region	Heat stress	↓ Yield; ↓ fruit set & photosynthesi s	↑ Flavonoids & phenolics, ↓ chlorophyll, membrane damage, altered sugar/carbohydrate metabolism	Heat-tolerant genotypes & osmoprotectants	[375]
Tomato	France	Heat stress	↓ Fruit set	Irregular ripening, fruit drop from style protrusion	Use of heat-tolerant genotypes	[85]
Tomato	Italy	Heat stress	-	↓ Fruit size; ↑ titratable acidity	Installation of semi-transparent/opaque PV panels above crop to shade and cool the soil, reducing irrigation demand	[376]
Tomato	Spain	Heat stress	−4% Total yield	↓ Fruit size, ↑ BER, ↓ firmness	Evaporative cooling (pad-fan)	[377]
Cucumb er	Cyprus	Heat stress and UV	↓ Yield	Yellowing and sunscald	Whitewash shading vs fan-pad evaporative cooling	[183]

					+ optimized irrigation regimes	
Sweet pepper	Italy	Heat stress and UV	↓ Fruit productivity	Physiological disorders	Use of plastic shading nets: 50% reflective Aluminet and 30% red Chromatinet;	[186]
Sweet pepper	Spain	Heat stress and UV	↓ Yield	↑Sunscald, ↓ fruit vitamin C and phenolics	Grafting ('Herminio' scion → Creonte rootstock); shading screen (Aluminet 40%) compared	[231]
Tomato	Spain	Heat stress and UV	↓31% (2010), ↓21% (2011) Fruit mass per plant	↓ Firmness, ↓ fruit size, ↑ fruit surface T→ faster quality loss	Fogging + plastic screenhouse (SFS)	[378]
Tomato	Spain	Heat stress and UV	-	Slight ↓ in fruit diameter and mass; delayed coloration	Use of roof-mounted flexible photovoltaic (PV) panels for shading and energy generation	[241]
Cucumber	Egypt	Heat and salinity stress	↓ Fruit set, ↓ marketable yield	↓ Chlorophyll, ↓ antioxidant status	Grafting onto tolerant rootstocks (VSS-61 F1, Ferro)	[225]
Cucumber	Egypt	Heat and salinity stress	↓ Marketable fruit yield	↓ Fruit firmness ↓ TSS,	Foliar application of silicon (200 mg/L), nano-Se, or H_2O_2	[222]
Tomato	Spain	Heat and salinity stress	↓ Total yield by ~30% under combined stress	↑ lycopene, ↑ lutein, ↑ TSS, ↑glucose/fructose, ↑ acidity, ↓phytoene, phytofluene, and phenolic compounds	—	[379]
Cucumber	Spain	UV stress	↓ Marketable yield	↓ Uniform fruit size and shape, physiological disorders (curling, scarring).	Passive heating: black plastic mulch + movable/fixed thermal screens	[343]
Cucumber	Spain	UV stress	↓ Yield	Fruit yellowing, ↓ size, and quality defects	Model-based irrigation aligned with radiation and VPD to maintain soil moisture	[373]

Tomato	Mediterranean region	UV stress	−14% to −19% total yield, −37% marketable yield,	↓ Lycopene ↓ antioxidants ↑surface damage / sunscald, ↓ color intensity and uniformity	UV-blocking polyethylene greenhouse film	[195]
Tomato	Greece	UV stress	↓ Marketable yield	Sunburn and cracking	White shading nets	[380]
Sweet pepper	Tunisia	Salinity stress	↓ Fruit number (−7 to −11%) & average fruit weight (−10 to –14%), ↓ yield by 14–43%	↓ Fruit set and quality	Use RDI (FI-MDI60), or DI-80 to maintain yield and control salinity	[236]
Sweet pepper	Turkey	Salinity stress	↓ Marketable yield	↓ Fruit set and quality	Foliar application of 2 mM silicon + 100 µM nitric oxide every 14 days under salinity	[369]
Tomato	Spain	Salinity stress	↓ shoot biomass and fruit yield; altered ion accumulation (Na^+, Cl^-); differences in cultivar tolerance	↑ TSS,↑ acidity, maintained firmness, ↑ antioxidant compounds	Use of different soilless culture systems (coconut fiber, perlite, rockwool)	[181]
Cucumber	Greece	Salinity and drought stress	↓ Yield	↓ fruit firmness, ↓nutritional quality and antioxidant activity	Foliar application of Si (K_2SiO_3 at 2.25 mM)	[367]
Sweet pepper	Turkey	Salinity and drought	Up to −60% yield	-	Water management and salinity control	[235]
Cucumber	Egypt	Drought	↓ Fruit number (–56%), ↓ fruit weight (–28%), ↓ total yield (–28%)	↑ Dry matter, ↓ TSS & vitamin C, ↑ electrolyte leakage, ↓ turgor	Foliar glycine betaine (6000 mg/L) over drought period	[381]
Cucumber	Egypt	Drought	↓ Fruit number per plant (–56%),	↓ Fruit size, ↓ diameter, ↓ volume,	Deficit drip fertigation + salicylic acid spray	[382]

			↓ yield (−28%), ↓ fruit weight and volume	↓ weight, ↓ leaf relative water content, ↓ turgor		
Sweet pepper	Italy	Drought	Dry matter & fruit yield ↓ by up to 23–33% under 60 % ET; ↓ up to 38–46% under 40 % ET	↓ Fruit number and size leading to lower marketability	Maintain irrigation at ≥60% ET; use deficit irrigation cautiously during non-critical growth phases	[383]
Sweet pepper	Spain	Drought	−19% to −55% yield	↑ Soluble solids, ↑ Phenolics, ↑ BER	RDI at 75% IWR during harvest	[47]
Sweet pepper	Spain	Drought	–33% total fruit production, ↓ marketable yield by –47% and –67%	↑ Unmarketable fruits due to small size, sunburn and BER	Maintain ET above 55% of available soil moisture (allowable depletion ~27 mm)	[220]
Tomato	Italy	Drought	↓≈15 % Marketable yield	↑ °Brix (~+10%), ↑ acidity, ↑ firmness, ↓ fruit size	Deficit irrigation	[48]
Tomato	Italy	Drought	↓40% Total yield	↑ °Brix, ↑ dry matter, ↑ acidity, ↓ fruit size	Tensiometer-based irrigation	[213]
Tomato	Spain	Drought	↓ Marketable yield	↑ Fruit cracking, ↓ β-carotene (−15 to –20%)	Misting/fogging systems	[384]
Tomato	Turkey	Drought	↓ Total and marketable yield	↑ Fruit dry matter and soluble solids	Use of drought-resilient Mediterranean tomato landraces in hydroponic (soilless) systems	[342]
Cucumber	Greece	VPD	↓ Yield; ↓ transpiration and CO_2 assimilation	↓ Turgor and slightly lighter green color	Shading + fogging systems	[11]

Table S3. Climate-induced responses on pest and disease pressure in greenhouse crops ranked by crop and pest/disease threat. The temperature (T)-related factors (heat stress, and high T) are considered together, without implying that they are interchangeable or equally stressful to plants. High T typically corresponds to an increase in vapor pressure deficit (VPD), which is also recognized as a climate change-associated stressor. BYI, before yellowing initiates; CMV, cucumber mosaic virus; FOL, *Fusarium oxysporum* f. sp. *lycopersici*; FORL, *Fusarium oxysporum* f. sp. *radicis-lycopersici*; IPM, Integrated Pest Management; RH, relative air humidity; ToBRFV, Tomato brown rugose fruit virus; TSWV, tomato spotted wilt virus; TYLCV, tomato yellow leaf curl virus.

Crop	Country/Region	Pest/Disease Threat	Climate Driver	Impact	Adaptation Strategy	Reference
Cucumber	Mediterranean region	CMV	Heat stress & wider seed trade	Distinct mosaic symptoms, fruit deformation; ↓ yield and quality	Certified virus-free seeds, apply stringent sanitation of tools/greenhouses, monitor plant health, removal of infected plants promptly	[385]
Cucumber	Greece	CMV	Heat & seed-borne survival	CMV and fruit deformation; virus persisted in soil >10 months, risking future crops	Certified virus-free seed, strict sanitation, detect via IC-RT-PCR, crop rotation	[386]
Cucumber	Israel	CMV	Heat stress; ↑ virus spread	Severe ↓ yield and fruit deformation; persistent infections via tools/soil	Virus-free compost and seeds, sanitation of tools and equipment, early detection, plant removal	[387]
Cucumber	Israel	CMV	Heat stress	Symptoms advance rapidly post-recovery under heat waves, vulnerability and potential ↓ yield/quality	Early symptom detection window (BYI stage) for timely rogueing/removal, apply T-based monitoring, implement hygiene, sanitation practices	[149]
Vegetables & ornamentals (e.g., tomato, pepper	Israel	CMV, whiteflies (*Bemisia tabaci*), thrips (*Frankliniella occidentalis*)	Heat and UV stress	↑ Virus/pest pressure; ↓ crop quality and marketability	Use of photoselective shade nets integrated with insect-proof greenhouse structures	[388]
Cucurbits, tomato	Mediterranean region (Italy, Turkey, Egypt)	CMV	Aphid-borne transmission enhanced by warm weather and intensive cropping	Widespread outbreaks in multiple crops; early infections cause yield loss, deformities	Integrated strategies: resistant cultivars, vector exclusion (e.g. nets), reflective mulches, removal of infected plants	[389]

Cucumber	Global (inc. Mediterranean)	Various viral & bacterial diseases (CMV, bacterial wilt)	Heat stress and RH ↑ disease spread	↑ Epidemic severity and spread ↑ disease incidence, ↑ rapid outbreaks, ↓yield and quality	Develop resistant cultivars, vector/bacterium monitoring, integrated sanitation and forecasting	[390]
Cucumber	Southern Tunisia	*Dacus frontalis* & *D. ciliatus*	Heat stress	Fruit infestation incidence ~21.8%; major risk for yield and marketability	Monitor fruit fly presence, develop targeted IPM, adjust cropping timing and fruit hygiene to reduce infestation	[391]
Sweet Pepper	Mediterranean Basin	TSWV	Heat stress and thrips activity	↑ Fruit deformation and ↓yield in susceptible cultivars	Breeding for TSWV resistance; thrips population control	[390]
Sweet Pepper	Mediterranean areas	Bacterial spot (*Xanthomonas* spp.)	Heat and RH; seed & irrigation spread	Necrotic leaf/fruits spots, ↓ yield up to ~30 % reported in warm regions	Integrated management: use disease-free seed, crop rotation, sprinkler alternatives, resistant varieties, bacteriophage/copper-based sprays	[392]
Sweet Pepper	Egypt	Bacterial leaf spot (*Xanthomonas vesicatoria*)	Heat and RH	↓ Fruit yield and quality parameters significantly (length, diameter, weight, number)	Foliar application of green-synthesized SiO_2 nanoparticles (150 ppm) + 5% antagonistic yeast (*Saccharomyces cerevisiae*); boosts plant defenses (PPO)	[393]
Sweet pepper	Mediterranean areas	Whiteflies (*Bemisia tabaci*, *Trialeurodes vaporariorum*); thrips (*Frankliniella occidentalis*); spider mites (*Tetranychus urticae*)	Heat stress	Consistent pest pressure	Implement augmentative biological control (predatory mites/bugs/parasitoids) timed based on crop phenology and pest cycles; integrate microbial agents; reinforce IPM to reduce chemical dependency	[394]
Sweet pepper	Egypt	Silverleaf whitefly (*Bemisia tabaci*)	Heat and RH; ↑ pests	↓ Fruit yield	Foliar application of green-synthesized SiO_2 nanoparticles?	[395]
Sweet Pepper	Israel	Pepper whitefly-borne vein yellows virus via *Bemisia tabaci*	Heat stress + insecticide-resistant	Fruit vein-yellowing, chlorosis, curling, discoloration, stunting, deformation, and	Timely whitefly (*B. tabaci*) control using biological agents/IPM; use of virus-free seed and resistant cultivars; monitoring	[396]

			whitefly spread	↓yield/marketability due to widespread infection	vectors and symptoms for early intervention	
Tomato	Algeria	Early blight: *Alternaria solani*, *A. linariae*, *A. grandis*, *A. protenta*	Heat & RH ↑ pathogen growth	Rot, concentric lesions (bullseye), sunscald due to defoliation, ↓ quality and yield	Use molecular diagnostics (PCR, RFLP) to accurately identify species and assess virulence, improve crop rotation and integrated disease management in Mediterranean tomato cultivation	[397]
Tomato, leafy greens, cucurbits	Italy	Fungal diseases promoted by poor air circulation (*Botrytis*, *Downy mildew*, *Pythium*)	High RH in closed environments during warm seasons	↑ Disease incidence due to moisture accumulation on leaves and flowers	**Improved natural and forced ventilation systems:** roof & side vents, insect-proof openings, fans, thermal screens	[344]
Tomato, cucumber, sweet pepper	Morocco, Tunisia	Soil-borne fungal and bacterial pathogens (*Fusarium spp.*, *Verticillium spp.*, *Pythium spp.*, *Rhizoctonia spp.*, *Ralstonia solanacearum*)	Warm T, intensive greenhouse cultivation	↑ Incidence of wilt and root rot diseases; ↓ yield and fruit quality	Soil solarization using transparent polyethylene to thermally inactivate soil pathogens	[398]
Tomato	Israel & Northern Italy	*Botrytis cinerea* (grey mold)	Heat and RH	In Israel: infection on leaves, stems, and fruit (rot and ghost-spot), ↓ marketable yield.<br>In Italy: severe fruit rot	Fungicide programs (iprodione/procymidone + thiram, tebuconazole mixes)<br>↓ disease by ~88%; *Trichoderma harzianum* T39 ↓ incidence by 31–82%; alternating biocontrol with fungicides	[152]
Tomato	France, Spain, Italy	Soil-borne fungal pathogens (*Fusarium spp.*, *Verticillium spp.*, *Pythium spp.*), nematodes	Warm/humid greenhouse conditions	↑ Root and vascular diseases; pressure on sustainable control options	Sulfur-based fumigation (e.g., sulfur dioxide, polysulfide formulations) as soil disinfectants post-methyl bromide	[399]
Tomato	Eastern Mediterranean Turkey	Fusarium crown and root rot (FORL) & Fusarium wilt (FOL race 3)	Heat and RH	↓ Yield under protected conditions	PCR-based diagnostics for early detection of FORL/FOL; implement resistant cultivars (with PCR-screened resistance);	[400]

					complement with soil sterilization and crop rotation	
Tomato	Algeria	FORL	Heat; ↑ pathogen aggression	↑ Disease incidence significantly higher at 28 °C vs 24 °C; crown and root rot; ↓ yield	Use genetic and VCG diversity data to guide breeding for resistance; implement soil and seed sanitation; apply biocontrol agents like *Trichoderma asperellum* and *T. ghanense*	[401]
Tomato	Mediterranean Basin	ToBRFV	Heat; ↑ virus spread	Severe outbreaks can lead to up to 100% ↓ yield due to fruit deformation, discoloration, and market rejection	Use of certified virus-free seeds, resistant cultivars (if available), strict hygiene, and vector control	[402]
Tomato	Eastern Mediterranean	TYLCV	Heat favoring whitefly vectors	↓ Yield and fruit quality due to viral infection	Use of TYLCV-resistant cultivars; vector control (whitefly)	[390]
Tomato	Cyprus	TYLCV	Heat stress favoring whitefly (*Bemisia tabaci*) activity	Severe early-season viral outbreaks; ↓ yield and fruit quality	Early planting to escape peak vector pressure, roguing of infected plants, insect exclusion structures, and field isolation; practices indirectly supporting resistance by reducing infection pressure	[403]
Tomato	Morocco	Whitefly (*Bemisia tabaci*), TYLCV	Heat stress climate-induced pest pressure	↑ Pest outbreaks under warming; initial overuse of pesticides; ↓ yield and fruit quality under unmanaged conditions	IPM using insect-proof nets (50 mesh), resistant cultivars, biocontrol agents	[404]
Tomato	Mediterranean Basin	*Tuta absoluta*	Heat; ↑pest growth	↓ Yield 50–100%	Implement integrated pest management: use mirid predators (*Nesidiocoris tenuis*, *Macrolophus pygmaeus*), egg parasitoids, Bacillus thuringiensis, sex-pheromone monitoring, and selective insecticides	[405]

Tomato	Spain	*Tuta absoluta*	Heat; ↑ pest growth	↑ Pest pressure and infestation levels in protected cultivation; ↓ yield and fruit marketability	Mass trapping, sex-pheromone monitoring, biological control (Macrolophus pygmaeus, Nesidiocoris tenuis), selective insecticides, and region-specific IPM programs	[406]
Tomato	Cyprus	*Tetranychus urticae*	Heat; ↓ suitability for tomatoes	Climate models predict 30–100 % ↓ of suitable irrigated areas for tomato	Expansion irrigated cultivation into newly suitable regions, Improvement of integrated pest management by adjusting release schedules of *P. persimilis* based on new climate data, Breeding heat- and mite-resilient tomato cultivars	[407]

Table S4. Estimated water and energy demands in greenhouse crops ranked by crop and country.

**Country**	**Crop**	**Water Demand (L m^{-2} d^{-1})**	**Cooling Energy Demand (kWh m^{-2} y^{-1})**	**Reference**
Mediterranean region	Cucumber	3.2 – 4.4	90 – 115	[187]
Greece	Cucumber	3.6 – 4.5	90 – 110	[187]
Greece	Cucumber	3.5 – 4.4	95 – 120	[183]
Turkey	Cucumber	3.7 – 4.6	Not reported	[210]
Turkey	Cucumber	3.8 – 4.5	115 – 140	[408]
Spain	Cucumber	3.5 – 4.3	85 – 105	[343]
Spain	Cucumber	3.3 – 4.5	Not reported	[409]
Tunisia	Greenhouse crops (e.g. cucurbits)	4.3 – 5.0	~85 – 100	[410]
Spain	Sweet Pepper	3.8 – 4.7	105 – 135	[411]
Spain	Sweet Pepper	1.6 – 2.3	9.3	[412]
Spain	Sweet pepper	1.8 – 2.0	Not reported	[220]
Spain	Sweet pepper	3.8 – 4.5	105 – 135	[413]
Mediterranean region	Tomato	3.6 – 5.1	90 – 160	[414]

Greece	Tomato	2.7 – 3.0	Not reported	[415]
Italy (Sicily)	Tomato		9	[416]
Spain	Tomato	2.9 – 3.4	269	[417]
Spain	Tomato	3.9 – 5.0	110 – 150	[418]
Spain	Tomato	4.1 – 5.4	105 – 135	[248]
Algeria	Greenhouse vegetables	3.8 – 4.6	Not reported	[419]
Greece (Crete)	Field & greenhouse crops	3.6 – 4.8	90 – 120	[420]
Turkey	Controlled-environment crops	3.6 –5.0	100 – 130	[421]
Italy (Puglia)	Greenhouse vegetables (e.g. tomato, lettuce)	Not reported	~85 –100	[217]
Italy (Sardinia)	Greenhouse tomato, lettuce, basil (substrate-based)	3.5 – 4.6	Not reported	[217]
Turkey (Izmir)	Tomato, cucumber, lettuce (greenhouse)	Not reported	128 – 156	[422]

Table S5. Structural innovations in greenhouse crops ranked by crop and country. The temperature (T)-related factors (heat stress, and high T) are considered together, without implying that they are interchangeable or equally stressful to plants. High T typically corresponds to an increase in vapor pressure deficit (VPD), which is also recognized as a climate change-associated stressor. BER, blossom-end rot; CMV, cucumber mosaic virus; DSSC, dye-sensitized solar cell; g_s, stomatal conductance; IR, infrared; NIR, near infrared; RH, relative air humidity; RWC, relative water content; UV, ultraviolet; WUE, water-use efficiency.

Crop	Country/Region	Structural Innovation	Climate Challenge	Outcome	Reference
Vegetables & ornamentals (cucumber, pepper, leafy)	Israel	Photoselective (colored) shade nets integrated with greenhouse/insect nets	UV and pest & disease pressure	↑ Vegetative vigor, ↑ yield, ↓pest/disease (CMV, whitefly, thrips)	[388]
Mixed greenhouse crops (e.g. cucumber, tomato, lettuce)	Italy (general EU focus)	Insulating clear plastic covers and IR-absorbing films	Heating and cooling energy demand	↓ Seasonal energy use by ~30%; ↓ leaf T; ↑ chlorophyll biosynthesis and light diffusion	[199]
Cucumber	France	Low-position canopy heating + forced fan ventilation & double screens	High energy demand + condensation risk	Climate more uniform, 17 % energy savings, ↓ condensation on fruits, yield maintained	[188]
Cucumber	Greece	Shading nets (35%, 50%)	UV & heat stress	Photosynthesis rate ↓ (–20% at 35%, –34% at 50% shading); ↓ transpiration and g_s	[44]

Cucumber	Greece	External shade nets (35% & 50%)	UV, heat stress & VPD	35% Shading ↓ peak air T by ~2–4 °C (but didn't maintain <30 °C); ↓ VPD, ↑ g_s, shading over 35–40%; ↓ production – thus optimal at ≤35% shading	[11]
Cucumber	Greece	Cooling systems: fan ventilation vs. fan-pad evaporative cooling	Heat stress & high VPD	Fan ventilation ↓ internal T differential, ↑ transpiration by 60%, greatly ↓ drainage (–95% effluent), maintained yield under soilless conditions with improved WUE at 0.24 L m^{-2} irrigation	[184]
Cucumber	Greece	Whitewash shading vs fan-pad evaporative cooling	Heat stress, high VPD, high water/energy use	Whitewash + ventilation maintained crop growth; ↑ transpiration (↓ nutrient outflow); similar WUE; no yield ↓; irrigation dense/performance unchanged	[183]
Sweet pepper	Italy, Mediterranean	External shading net (35% light reduction)	Heat and UV stress	Shading improved microclimate yield maintained or slightly ↑; no adverse effects on fruit color; phenolic & antioxidant levels stable	[423]
Sweet pepper	Spain	Hybrid passive system: evaporative screening + water-filled sleeves + movable shading/thermal screen	Heat stress	Improved VPD during hot periods; ↑ leaf area index; ↑ early vegetative growth & marketable fruit, +25 % yield; –8 % irrigation demand; +20 % irrigation water efficiency	[411]
Sweet pepper	Spain	Reflective aluminized shade screens (40% and 60%)	UV and heat stress	40% shading (T40); ↑ yield by +1.26 kg/m² vs unshaded; ↓ sunscald and unmarketable yield; 60% shading maintained yield but didn't improve it	[110]
Sweet pepper	Spain (Mediterranean)	Whitewash roof shading + fogging + forced ventilation	Heat stress and high VPD	Whitewash & fogging effectively ↓max T /VPD; whitewash had highest WUE (16.2%), fog (29.2%); whitewash most profitable	[413]
Sweet pepper	Spain (Mediterranean)	Passive climate control system:	Extreme T fluctuations	Enhanced fruit nutritional quality: ↑ ascorbic acid,	[128]

		water-filled sleeves + movable screens + thermal screens		phenolics, soluble solids; ↓ heat damage during spikes	
Sweet pepper	Turkey	Drip irrigation regimes with saline water	Salinity and drought	Optimal water productivity achieved at 50% Class A pan-evaporation irrigation; salinity threshold ~1.43 dS/m with ~11.1% yield ↓ per unit dS	[219]
Tomato	France (Research setting likely France/Ivry-sur-Seine)	Diurnal T rise management via greenhouse climate control	Heat stress	Faster fruit maturation (-5 days), ↓ fresh weight (-30 %), dry matter (-30 %), lower firmness, affected sugar/acid balance; fruit quality impacts could be partly mitigated	[424]
Tomato	Greece	Semi-transparent dye-sensitized solar cell (DSSC)–covered greenhouse	UV + dual goal of electricity generation and crop productivity	+6–26% ↑ in bioactives (ascorbic acid, lycopene, β-carotene, total carotenoids) and +5–10% antioxidant activity	[194]
Tomato	Greece (Crete)	Climate-smart shade nets (white, pearl, photo-selective)	Heat stress, high radiation	↓ BER; ↑ lycopene accumulation; ↓ sunscald; ↑ WUE and marketable yield	[420]
Tomato	Italy	DSSAT crop model simulating future climates	Heat stress and drought	Projected 15% yield ↓; offset requires +85–110 mm irrigation & +20–30 kg N/ha; modest <10% gains in water and N use efficiency under adaptation	[425]
Tomato	Spain	Diffuse film covers	Heat and UV stress	↑ Light distribution, ↓ canopy T, ↑ yield	[377]
Tomato	Spain	Flexible rooftop photovoltaic panels (~9.8% roof)	UV + need for renewable energy integration in semi-arid greenhouses	No effect on total or marketable yield; slight ↓ in fruit diameter and mass and delayed color development; fruit remained marketable	[241]
Tomato	Spain	Integration of ridge and side ventilation openings	Heat stress	5 °C reduction in internal T; 18% yield increase	[190]

Tomato	Turkey	Drip irrigation with saline irrigation water (0.7–7.5 dS m^{-1})	Salinity	↑ soil salinity, ↓ evapotranspiration, leaf area index, and marketable yield (up to 55–60% ↓ at 7.5 dS m^{-1}); g_s unchanged	[426]

Table S6. Water management innovations in greenhouse crops ranked by crop and country. The temperature (T)-related factors (heat stress, and high T) are considered together, without implying that they are interchangeable or equally stressful to plants. High T typically corresponds to an increase in vapor pressure deficit (VPD), which is also recognized as a climate change-associated stressor. AI, artificial intelligence; BER, blossom-end rot; DM, Dry Matter; DSS, Decision Support System; EC, electrical conductivity; ET, evapotranspiration; EU, European Union; g_s, stomatal conductance; IoT, Internet of Things; LCA, life cycle assessment; MSW, municipal solid waste; OM, organic matter; PV, Photovoltaic; RDI, regulated deficit irrigation; TA, Total Antioxidants; TDR, time domain reflectometry; TI, thermal index; TSS, total soluble solids; UV, ultraviolet; VPD, Vapor Pressure Deficit; WUE, water-use efficiency.

Crop	Country/Region	Water Management Practice	Climate Challenge	Outcome	Reference
Greenhouse vegetables	Europe (Mediterranean & beyond)	Adapted FAO56 ET, soil/substrate moisture sensors (tensiometer, dielectric, capacitance), plant sensor (stem diameter, canopy T), and DSS-based irrigation scheduling	Drought, nutrient leaching	↑ WUE, ↓nitrate leaching; sensor adoption ranges from 15% to 95% across EU	[427]
Greenhouse vegetables (tomato, cucumber, pepper etc.)	Mediterranean region	Conservation tillage, drainage water reuse, sensor-based & timed irrigation systems	Drought & resource efficiency	~38 % less water/energy use, ~62 % environmental benefit, ~13 % LCA gain per ton	[273]
Tomato, Pepper, Cucumber	Spain (Mediterranean greenhouse)	Drip irrigation scheduled by ET/Kc, whitewash, sand mulch, PrHo ET calculator software	Heat & drought	Seasonal ET 170–371 mm; Kc values: 0.2→1.4→1.0; WUE 15–36 kg/m^3; RIS ~1.6 (cucumber)	[409]
Greenhouse vegetables	Portugal	Intelligent DSS platform (ID3SAS) with sensor integration & data-driven scheduling	Drought	Improved irrigation efficiency; adaptive, real-time decisions; reduced water & energy inputs	[428]
Greenhouse vegetables	Algeria	Reuse of agricultural drainage water and municipal wastewater for crop irrigation; assessment of water quality and planning of filtration infrastructure	Drought	Improved irrigation efficiency	[429]

Leafy vegetables, herbs, tomatoes	Israel	Hydroponic & recirculating closed-loop systems	Drought	↓ Water & fertilizer use; ↑ yield/unit area; enhanced hygiene potential; microbial risk requires monitoring	[430]
Mixed crops (field & horticultural)	Lebanon	Integrated water resource planning; reuse of treated wastewater & drainage	Drought	Sustainable irrigation	[431]
Olive orchards, field vegetables	Tunisia	Small-scale desalination units for irrigation	Drought	Desalination (reverse osmosis) produced freshwater (<500 mg/L TDS); water cost 0.30–0.50 €/m³; supported crop yields	[432]
Not specific	Turkey	Grid-connected PV-powered small-scale desalination plants	Drought	Solar potential strong (1,775 kWh/m²·yr); PV desalination feasible; pilot RO units (~700k m³/yr) operational for agriculture	[433]
Cucumber	Algeria	Reuse of treated drain water in greenhouse irrigation	Drought	Improved life cycle sustainability, potential trade-offs (fertilizer & energy)	[185]
Cucumber	Egypt	Pulse drip irrigation (3 pulses/day) + pulse fertigation (8/season)	Drought	↑ WUE to 5.6 kg m⁻³, yield ~34 t ha⁻¹, ↑ vitamin C & TSS	[219]
Cucumber	Italy	Root-zone sensors (tensiometer, dielectric, capacitance) + automated scheduling	Drought	Improved water scheduling, ↓ over-irrigation, maintained yield/quality	[246]
Cucumber	Lebanon	ET-based soil moisture scheduling + continuous fertigation (SEALACOM)	Drought	Water saved ≈105 mm/season; +20% yield; WUE >60%; N use efficiency doubled/tripled vs traditional	[434]
Cucumber	Palestine	Deficit irrigation (70% ET)	Drought	↑ WUE, ↑ yield 24% vs TI	[133]
Cucumber, tomato	Mediterranean region	Sustainable irrigation (pressurized, deficit, non-conventional water, AI-enabled)	Drought	↑ WUE	[182]
Cucumber, tomato	Semiarid Mediterranean	Energy-focused innovations (e.g. sensor-integrated water control, shading, DSS)	Heat stress & drought	↑ WUE	[184]

Cucumber, tomato	Italy	Drip irrigation regulated by tensiometer at –100 vs –400 hPa (tomato) and –100 vs –300 hPa (cucumber)	Drought	Tomato: –35 to –46% water, +14–65% WUE; –400 hPa treatment: 40% yield ↑, but TSS +41%, DM +45%, TA +59%; Cucumber: –49 to –42% water, +73–96% WUE; DM +8%, yield stable	[213]
Pepper	Mediterranean	Grafting onto stress-tolerant rootstocks	Drought & salinity stress	↑ Tolerance—maintained photosynthesis and yield under stress	[435]
Pepper	Spain (SE Mediterranean)	Drip fertigation with increasing salinity (2.5–7.5 dS/m)	Salinity stress	Yield ↓ 37% at 5.5 dS/m; marketable yield ↓ 47%; BER ↑70%; ECsst ≈ 5 dS/m; physiological decline per	[208]
Pepper	Spain (Almería)	RDI (~600 m^3 ha^{-1} reduction) + seaweed & microbial biostimulants	Drought	WUE ↑ 17–34%, yield maintained or ↑; ↑root uptake & soil enzymatic activity	[436]
Pepper	Spain (Mediterranean greenhouses)	Automated fertigation using electrotensiometers at soil matric potential thresholds (−10 vs −20 kPa)	Drought	−10 kPa threshold ↑ yield and WP (~24 kg m^{-3}); −20 kPa ↓ water by 22% but lowered yield; no drainage observed	[212]
Tomato	Greece	Roof rainwater harvesting with tank storage (100–290 m^3/1,000 m^2), balancing dry-season water demand	Drought	Tomato: up to 100% demand reliability (290 m^3);	[211]
Tomato	Greece	Drip fertigation using treated wastewater + municipal solid waste compost amendment	Low-fertility soil, brackish/poor-quality water	≥20% MSW: ↑ biomass; 40% MSW: highest yield; fertigation boosted yield at 5–10% MSW; TWW maintained yield + quality; soil OM & nutrients improved	[437]
Tomato	Greece	Drip irrigation with saline water (ECw 1.1 vs 3.5 dS/m); TDR sensors + SALTMED model for moisture and leaching	Salinity, high VPD and heat stress	High-ECw irrigation, ↑ root-zone salinity	[438]
Tomato	Greece	Hydroponic closed-loop greenhouse system	Drought	↑ Water & fertilizer efficiency via recirculation; economically viable within 5 years	[439]
Tomato	Italy (Tuscany)	Low-cost IoT smart drip irrigation	Heat stress	Water savings, yield maintenance	[180]

Tomato	Spain	Irrigation with continuous/intermittent ozonated water	Salinity stress	↑ Photosynthesis, ↓ g_s, ↑ WUE; yield & quality maintained; soil health supported	[181]
Tomato	Spain	Smart, plant water-use triggered irrigation (thresholds: 0.4 L/plant for FR1; 0.8 L FR2; 1.2 L FR3)	Heat stress	At 32 °C FR2/FR3 saved 16%/33% water vs FR1; similar yield for FR2, FR1; FR3 ↓ yield; plant water potential & gas exchange monitored	[181]

Table S7. Variety selection and agronomic practices for climate resilience in greenhouse crops ranked by crop and country. The temperature (T)-related factors (heat stress, and high T) are considered together, without implying that they are interchangeable or equally stressful to plants. High T typically corresponds to an increase in vapor pressure deficit (VPD), which is also recognized as a climate change-associated stressor. BER, blossom-end rot; ET, evapotranspiration; MAS, marker-assisted selection; PV, Photovoltaic; RDI, regulated deficit irrigation; Si, silicon; TSS, total soluble solids; WUE, water-use efficiency.

Crop	Country/ Region	Stress Factor	Yield Impact	Quality Impact	Strategy	Reference
Cucumber	Cyprus	Heat stress and high VPD	↓ yield	↓ Canopy T and VPD, preserving photosynthetic activity and preventing quality disorders	Greenhouse cooling systems evaporative fog cooling and roof whitewash shading	[184]
Cucumber	Egypt	Heat and salinity stress	↓ Yield in ungrafted plants; grafting onto tolerant rootstocks restored marketable yield	Maintained fruit set (lower flower abortion) and fruit TSS under stress; no loss of firmness with appropriate rootstocks	Grafting onto heat- and salt-tolerant rootstocks (e.g. *Cucurbita* hybrids 'Ferro' and 'VSS-61 F1')	[225]
Cucumber	Egypt	Heat stress	Enhanced yield stability under heat; identified superior lines	Maintained fruit size, TSS, and texture under stress	Screening Egyptian germplasm; selecting diverse parents for hybrid development targeting heat tolerance	[65]
Cucumber	Greece	Drought and salinity stress	↓ plant growth and ↓yield potential (↓fruits)	Treated plants maintained leaf water content and photosynthesis, and produced firmer fruits with delayed senescence	Silicon application (2.25 mM potassium silicate) to enhance drought and salt tolerance	[367]

Cucumber	Lebanon	Drought and salinity stress	Traditional irrigation caused salinity buildup and yield ↓	↑ WUE and water productivity	Drip fertigation and plastic mulch to improve soil moisture and reduce salt accumulation	[434]
Cucumber	Palestine	Drought	A moderate deficit regime (70% crop ET replacement every irrigation) ↑ total yield (~59.5 t/ha) than full irrigation (~55–57 t/ha), while a suboptimal farmer/tensiometer schedule, ↓ yield (~45 t/ha)	The 70% ET regime improved fruit number per plant without harming fruit size	RDI irrigating at ~70% of full crop water requirement in a controlled schedule	[133]
Cucumber	Tunisia	Drought	↑ WUE, ↓ fruit quality defects related to water stress (e.g. tip burn).	↑WUE and ↓ fruit quality defects	Subsurface drip irrigation (placing drip lines ~15–30 cm deep)	[363]
Cucumber	Turkey	Drought	Deficit irrigation (75% ET) ↓ yield ~30% (from ~128 to 90 t/ha) compared to full irrigation	↑ WUE improved as ↑ irrigation; highest WUE (~42–56 kg yield per m^3) under the most water-limited regime	Optimizing irrigation scheduling based on evapotranspiration, (~80–85% of full irrigation)	[210]
Sweet Pepper	Greece	Heat stress	Yield varies with cultivar and grafting	Improved WUE and nutrient use efficiency	Use of tolerant cultivars and grafting techniques to optimize hydroponic performance	[440]
Sweet Pepper	Italy	Heat stress and UV	↑ yield (~10–15% vs. no shade)	↑ antioxidant content (carotene, vitamin C, tocopherol)	Shade net (35% blackout) over greenhouse	[423]
Sweet Pepper	Italy	Salinity stress	Higher yield under salt stress	Physiological traits supporting WUE	Selection of chloride-exclusion landrace; potential MAS QTL	[441]
Sweet Pepper	Spain	Heat stress	↑ marketable fruit than ungrafted during heat	↑ fruit set, reduced leaf damage, better chlorophyll retention	Grafting onto heat-tolerant rootstocks	[361]

Sweet Pepper	Turkey	Heat and salinity stress	↑yield by ~16.5% under salt stress	↓ stress indicators, ↑stomatal function and fruit quality	Si nutrition + NO foliar spray every 14 days	[369]
Tomato	Greece	Heat and drought stress	Some hybrids maintained >90% of greenhouse yield under low-input conditions	Resilient hybrids- maintained size, Brix, lycopene, vit. C	Choose hybrids tested for low-input resilience	[365]
Tomato	France	Heat stress	Most cultivars lost yield; only few retained partial fruit set	2/11 cultivars had some viable pollen; rest collapsed	Select cultivars maintaining pollen under heat	[358]
Tomato	France	Heat stress	Identified genotypes with stable yield under heat; 69 plasticity QTLs for 11 traits; candidate genes related to pollen viability and fruit set	Maintains fruit set and quality under heat conditions; expression of ROS-scavenging genes, HSPs, HSFs	Genome-wide QTL mapping (MAGIC & core-collection); transcriptome profiling of floral tissues; candidate gene identification	[230]
Tomato	Israel	Heat stress	Up to 100% yield ↓ in sensitive lines; tolerant lines retained ~50%	Pollen viability ↓ in most, some tolerant lines stable	Select heat-tolerant lines with viable pollen under heat	[360]
Tomato	Israel	Heat, drought, salinity stress	Multi-trait improvement (flowering, fruit development, stress resilience)	Genome-wide multi-targeted CRISPR library (15,804 sgRNAs targeting ~10 gene families) + CRISPR-GuideMap barcode tracking	Identified ~100 mutant lines with altered fruit shape, flavor, nutrient uptake, pathogen response; enables precision breeding to target functional redundancy and stress-adaptive traits	[442]
Tomato	Italy	Drought	~10–12% ↓ yield at 75% ET; 30–35% at 50% ET	↑°Brix at 75% ET; poor quality at 50% ET (rot, small fruit)	Use regulated deficit irrigation at ~75% ET	[48]
Tomato	Italy	Heat and drought stress	6–11% ↓ yield projected by mid-century without adaptation	Slight TSS under heat; quality ↓ if water-stressed	Shift planting, efficient irrigation, heat-tolerant varieties	[425]
Tomato	Italy	Chronic combined	Maintained fruit yield and plant biomass	Preserved photosynthetic efficiency, reduced	Selection and physiological characterization of a	[237]

		water and heat stress	under prolonged stress	ROS damage, improved WUE	novel genotype with dual stress resilience	
Tomato	Saudi Arabia	Heat stress	~0% yield in sensitive lines; tolerant lines yielded 20-30%	Tolerant lines had stable pollen, meiosis; HSP genes	Use genetic markers to breed heat-tolerant lines	[356]
Tomato	Spain	UV & partial shading	2–8% lower under PV shading vs. full sun	↑ under PV (less sunburn/rot); sugar & firmness stable	Semi-transparent PV panels for co-generation and shading	[443]
Tomato	Spain	Salinity, drought, heat, oxidative stress	Maintained yield; improved fruit set and biomass under stress	Enhanced carotenoids, tocopherols, sugars, and acids; improved flavor	Functional genomics (e.g., SlCBL10, SlDEAD39); QTL analysis, gene function studies, and TILLING.	[444]
Tomato	Spain	Heat stress	~15–20% ↓ yield under unmitigated heat; minimal loss with treatment	Treated plants retained firmness, acidity, flavor	Apply oligosaccharin foliar sprays as heat biostimulant	[362]
Tomato	Turkey	Salinity	~20–30% ↓ at 4 dS/m; severe at 8 dS/m	↓ Size; BER, slight TSS	Salt-tolerant varieties, <4 dS/m drip irrigation, breeding	[426]
Tomato	Turkey	Soil degradation and nutrient imbalance	Improved yield stability across seasons	Enhanced soil microbial activity and structure	Pre-plant crop diversification and tomato-based rotation for soil health restoration	[445]
Tomato	Turkey	High T during fruit development	Maintained yield under heat stress	Elevated bioactive compounds (phenolics, carotenoids, antioxidants)	Selection and characterization of heat-tolerant genotypes with enhanced bioactive profiles	[446]

Table S8. Adaptation strategies for climate resilience in greenhouse crops ranked by domain. AI, Artificial Intelligence; DSS, Decision Support System; IoT, Internet of Things; IPM, Integrated Pest Management; NFT, nutrient film technique; PCMs, Phase-Change Materials; PV, Photovoltaic; RH, relative air humidity; T, temperature; UV, ultraviolet; WUE, water-use efficiency.

**Domain**	**Strategy**	**Objective**	**Example/Technology**
Structure	Reflective screens	Reduce radiation & heat load	Aluminized shade nets
Structure	Roof-integrated PVs	Renewable energy generation	Building-integrated PV systems

Water	Moisture sensors	Irrigation precision	Tensiometers, capacitance sensors
Water	Water reuse systems	Minimize freshwater use	Recirculating hydroponics, UV-treated wastewater
Water	Rainwater harvesting	Alternative water source	Gutter systems, collection tanks
Crop	Heat-tolerant hybrids	Yield stability	‘SV8591’ tomato, local pepper landraces
Crop	Grafting on tolerant rootstocks	Enhance stress tolerance	Tomato on *S. habrochaites* rootstock
Crop	Adjusted sowing/harvest periods	Avoid heat peaks	Seasonal shifting in Crete and southern Italy
Energy	Renewable integration	Carbon footprint reduction	Solar PV panels, geothermal loops
Energy	Thermal screens and PCMs	Reduce energy load	Internal shading, phase change materials
Energy	Smart energy controllers	Real-time optimization	AI-based energy management platforms
Climate Control	Evaporative cooling systems	T reduction	Pad-and-fan systems, high-pressure foggers
Climate Control	Insect-proof ventilation	Pest exclusion	Fine mesh nets with forced airflow
Climate Control	Dynamic shading	Regulate light exposure	Movable thermal screens, chromatic shading
Soil/Substrate	Soilless cultivation	Improve WUE and control	Rockwool, cocopeat, NFT systems
Monitoring	Integrated sensor networks	Real-time climate and crop tracking	IoT-connected weather stations and plant sensors
DSS/Automation	DSS	Optimize irrigation/fertigation/control	Hort@, iGreenhouse, AgriSens
Pest & Disease	Biocontrol and IPM	Reduce pesticide use	*Macrolophus pygmaeus*, bumblebee pollination
Postharvest	Climate-adapted handling protocols	Preserve quality under stress	Cold chain systems with RH control
Training	Farmer capacity building	Enhance adaptation skills	Online platforms, demo farms, extension services
